# Mental Health Literacy for Supporting Children: A Systematic Review of Teacher and Parent/Carer Knowledge and Recognition of Mental Health Problems in Childhood

**DOI:** 10.1007/s10567-023-00426-7

**Published:** 2023-02-10

**Authors:** Catherine L. Johnson, Maxine A. Gross, Anthony F. Jorm, Laura M. Hart

**Affiliations:** 1 Centre for Mental Health, Melbourne School of Population and Global Health, The University of Melbourne, Level 5, 207 Bouverie Street, Carlton, VIC 3010 Australia; 2 School of Psychology and Public Health, La Trobe University, Bundoora, VIC Australia; 3 Telethon Kids Institute, Adelaide, SA Australia

**Keywords:** Mental health literacy, Child, Parents, Teachers, Mental disorders, Early intervention

## Abstract

The level of mental health literacy (MHL) in adults who work with or care for children is likely to influence the timeliness and adequacy of support that children receive for mental health problems. The aim of this study was to systematically review the literature on mental health literacy for supporting children (*MHLSC*, recognition/knowledge) among parents and teachers of school aged children (5 to 12 years old). A systematic search was conducted for quantitative studies published between 2000 and June 2021 using three databases (MEDLINE, PsycINFO and ERIC) and relevant citations reviewed in Scopus. To be included, studies needed to measure at least either ‘mental health knowledge’ or ‘recognition’. Synthesis proceeded according to study design, adult population, child MHP, then MHL outcome. Study quality was assessed using AXIS. 3322 documents were screened, 39 studies met inclusion criteria. 49% of studies examined teachers’ knowledge or recognition of ADHD; only five studies reported on parent samples. Synthesis found a nascent field that was disparate in definitions, methods and measures. Little research focussed on knowledge and recognition for internalizing problems, or on parents. Methods used for measuring knowledge/recognition (vignette vs screening) were associated with different outcomes and the quality of studies was most often low to moderate. Adults appear to have good recognition of childhood ADHD but their knowledge of internalizing disorders is less clear. Further research is required to develop standard definitions and validated measures so gaps in MHLSC can be better identified across populations who have a role in supporting children with their mental health.

**Supplementary Information:**

The online version contains supplementary material available at 10.1007/s10567-023-00426-7.

Many mental health problems (MHPs) have their onset in childhood or are first detected before the age of 14 (Solmi et al., 2021). These include the most commonly diagnosed disorders of childhood: anxiety disorders and attention-deficit hyperactivity disorder (ADHD), but also others such as autism spectrum disorders (ASD) and eating disorders (Bitsko et al., 2022; Lawrence et al., 2015). Good mental health in the early years of life provides a solid basis for positive developmental trajectories into young adulthood and beyond (The Royal Australian & New Zealand College of Psychiatrists, 2010). Conversely, MHPs experienced in childhood, if left undetected and untreated, can lead to immediate and long-term consequences for individuals and their families, as well as increased burden on the health care system (Bittner et al., 2007; Castagnini et al., 2016; Copeland et al., 2015). Effective treatments are available and early intervention is key to mitigate the effects of MHPs on child development and functioning, yet many children do not access the help they need (Mulraney et al., 2020; Simon et al., 2015). Population-level surveys around the world have estimated that somewhere between 1 in 10 (10%) and 1 in 7 (14%) of children experience a MHP in any given year, however, only about half of these receive appropriate services (Bitsko et al., 2016; Johnson et al., 2016; Lawrence et al., 2015; Lu, 2017; Sadler et al., 2018). This may be, in part, due to childhood MHPs being poorly understood and recognized within the general populations that live and work with children (Tully et al., 2019).

*Mental health literacy* (MHL) is a concept first proposed by Australian researcher Anthony Jorm and colleagues in the late 1990s. Jorm et al. defined MHL as “the knowledge and beliefs about mental disorders that aid their recognition, management or prevention” (Jorm et al., 1997, p. 182). Population-based research on MHL is well advanced (Furnham & Swami, 2018) and has led to the dissemination of effective public health interventions to improve public knowledge, attitudes and support skills towards those experiencing MHPs (Morgan et al., 2018; Reavley & Jorm, 2012b). Jorm’s, 2000 definition included six sub-components of MHL (see Fig. 1). Recognition and knowledge of MHPs are the most commonly and widely measured sub-components of MHL in the adult MHL literature (Mansfield et al., 2020; O'Connell et al., 2021; Wei et al., 2015). It is important to note that MHL encompasses not just conceptual knowledge of MHPs, but knowledge that is expected to increase the likelihood of action to benefit one’s own mental health or that of others (Jorm, 2012).

**Fig. 1:**
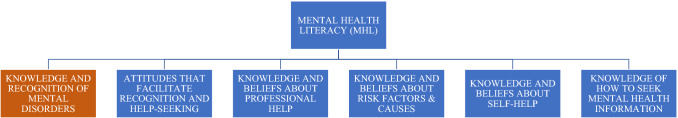
Mental health literacy and sub-components

Despite the advancement of the field of adults’ and youths’ MHL, our understanding of adults’ knowledge about the mental health of children (i.e. *mental health literacy for supporting children*, MHLSC) remains less developed. MHLSC is important to distinguish from MHL for adult- or adolescent-onset MHPs because there are fundamental differences in how presentation, management and prevention of MHPs occur across the lifespan. For example, child-onset MHPs often present differently to adolescent or adult-onset disorders, meaning that recognition of MHPs may require different types of knowledge (Fogarty & Mensah, 2021; Kessler et al., 2007).

Preliminary research on MHLSC suggests adults who have knowledge of child MHPs sufficient for problem recognition are more likely to endorse appropriate professionals as sources of help, seek out information on how to manage MHPs and access mental health services, than those who do not recognize child MHPs of concern. Thus, this early research points to connections between knowledge of child MHPs, recognition of child MHPs and the help-seeking pathway (Cormier et al., 2020; Oh & Bayer, 2015; Ohan et al., 2008a, b; Ryan et al., 2015; Thurston et al., 2015). This may be because children are unlikely to independently initiate help-seeking and require the support of a gatekeeper adult, who must have the knowledge to recognize a problem and that seeking help is necessary (Stiffman et al., 2004). By contrast, for adolescents and adults, recognition, knowledge and prevention may be individually driven and involve far fewer stakeholders in the steps of the pathway to professional help.

Parents, carers or guardians (henceforth referred to as ‘parents’), by virtue of the relationship they have with children, have been identified as those best placed to act as supports for children with MHPs (Johnco et al., 2019). In addition, in the early school years (ages 5 to 12 years), students spend a significant proportion of their time in classrooms with the same teachers and support staff, who teach across curriculum. Therefore, teachers’ and parents’ appropriate recognition of MHPs can be a vital step in the promotion of effective help-seeking or symptom management (Miller et al., 2019).

Reviews to date have focussed on the MHL of parents of both children *and adolescents*. For example, Hurley et al. (2020) predominately reviewed studies that examined the MHL of parents of adolescents, providing little insight into the distinctly different gaps in knowledge that may exist for parents and teachers of children (i.e. those aged 12 years or under, who would normally attend primary/elementary or middle school). No comprehensive reviews of teachers or parents’ knowledge of MHPs in *children* currently exist (Tully et al., 2019). Understanding the strengths and weaknesses of parents’ and teachers’ knowledge of MHPs in childhood could provide important insights for population mental health researchers aiming to design interventions to improve MHLSC in these populations (Splett et al., 2019).

The aim of this review was to systematically review and describe the available quantitative evidence for parents and teachers’ initial recognition of, or knowledge about, child MHPs, as two sub-components of the broader concept of MHLSC. Risk of bias analyses were conducted and implications for future population mental health research were considered.

## Method

This review followed the PRISMA 2020 guidelines (Page et al., 2021). The protocol was registered with PROSPERO (Gross et al., 2020) and updated prior to data extraction as needed.

### Eligibility Criteria

To be included in this review, studies needed to measure parents’ or teachers’ knowledge or recognition (whether or not these were conceptualized as a part of ‘mental health literacy’ within the publication) of MHPs (either broadly or by specific diagnosis) in children aged 5 to 12 years. Studies that measured additional sub-components of MHLSC (e.g. attitudes influencing recognition, or help-seeking; beliefs about professional sources of help, about causes and risk factors, or self-help interventions; and seeking information about MHPs) were also included, but these did not form inclusion criteria, so were only considered within papers that measured one of the two primary MHLSC components (recognition or knowledge). All other specific inclusion and exclusion criteria can be found in Supplementary File A.

### Information Sources

Three electronic databases—PsycINFO (Ovid), MEDLINE (Ovid) and ERIC (EBSCO)—were searched by the researchers in August 2020 (and updated in June 2021) for studies that met the inclusion criteria. In addition, Google Scholar was searched using the same key search terms. Any key studies or systematic reviews identified in the search were also then located in Scopus and a backwards citation search was performed to cross check the search strategy.

### Search Strategy

A search strategy was developed by two authors in consultation with the wider research team. Search terms for each of the concepts were initially developed by compiling relevant terms used in other systematic reviews (e.g. Hurley et al., 2020) as well as the key components of MHL as conceptualized by Jorm (2000) and expanded upon above. Once search terms were determined, they were mapped onto the relevant search language for each of the databases. The final search protocol and the mapped terms can be found in Supplementary File B.

The search strategy was first piloted and ran in July and August of 2020 and then updated in June 2021 to include relevant papers published between August of 2020 and June 2021.

### Selection Process

Study citations from the search were downloaded into Endnote (The Endnote Team, 2013), then exported to Covidence (Covidence Review Software, 2019). After removing duplicates, paper title and abstracts were independently screened by two authors (CJ and MG). Those meeting inclusion criteria were then independently double-screened as full text papers by two authors (CJ and MG). Any disagreement between the authors was discussed at team meetings and if needed, a third reviewer (LMH) helped to determine consensus.

In order to succinctly review the literature, we decided to review only the 38 quantitative studies (longitudinal and cross-sectional surveys, observational/experimental studies) and 1 mixed-methods study uncovered in the search. An additional 28 qualitative studies (interviews, focus groups, open-ended surveys) were uncovered, but were not well suited to measuring levels of knowledge or recognition—rather, they were better suited to exploring separate sub-components of MHL—such as the attitudes and beliefs underpinning recognition and help-seeking. Although related, this additional information provided by the qualitative papers was considered out of scope, given the focus of this study.

Similarly, our search uncovered seven studies reporting on interventions designed to improve MHLSC among parents or teachers. As this review aimed at understanding the relative strengths and weaknesses of adult’s MHLSC, the search strategy used was not designed to focus specifically on interventions. Thus, it was considered likely that additional specific searches for training or education would elicit more studies on this topic and this was outside the scope of the current research. The seven intervention papers were therefore excluded from the current review. At the step of full text screening, papers were categorized as interventions, quantitative, qualitative or mixed methods, and only those with quantitative or mixed designs were selected for inclusion.

### Data Collection Process

Data were extracted according to a pre-determined, structured template designed by one of the researchers (CJ) within Covidence, which included the fields: study author, year, country, sample size and characteristics, MHP focus, aims, measures used, relevant knowledge/recognition outcomes, other MHLSC outcomes, covariates examined with regard to knowledge/recognition and key results. Outcomes were coded under one of the six MHL sub-components outlined in Fig. 1. Extraction was first performed by the first author then checked for reliability by having the second author extract a random selection of 10 papers, while coding was performed by both authors together. Conflicts in data extraction and coding were resolved by discussion until consensus was reached. Data extracted on study results included any between or within-group comparisons, as well as confidence intervals (if available) and relevant *p* values (see Supplementary File C). Meta-analysis was not conducted due to the high heterogeneity of measures and outcomes.

### Study Quality Assessment

All studies were critically appraised using the Appraisal of Cross-Sectional studies (AXIS) checklist and accompanying guidelines (Downes et al., 2016). AXIS is cited in systematic and meta-analytic reviews of health research and is designed for assessing the quality of non-randomized, cross-sectional studies. AXIS consists of 20 questions. For each question, 1 point is awarded for a ‘yes’ response, whereas 0 points are awarded otherwise (i.e. a ‘no’ or ‘cannot determine’ response). Based on the total score as a percentage, the studies were categorized into three groups: high quality (≥ 80%), moderate quality (> 60% but < 80%) or low quality (≤ 60%). Assessment of quality was conducted by the first author and a random selection of 50% of the papers was assessed by the second author. There was an acceptable level of agreement between raters (*κ* = 0.84), and any disagreement was discussed until consensus was reached.

### Synthesis Methods

Although most studies reviewed used a cross-sectional survey design, there was significant heterogeneity in the outcome measures used and results reported. Therefore a modified narrative synthesis method, as outlined by Popay et al. (2006), was used instead of quantitative data synthesis. This process involved four steps: (1) developing a theoretical model, (2) developing preliminary synthesis, (3) exploring relationships in the data and (4) assessing the robustness of the synthesis product. The theoretical model guiding the synthesis was Jorm’s (2000) MHL framework, which was chosen prior to conducting the systematic search. As part of Step 2, the first author-developed a table of key study characteristics from the data extraction templates. This tabular summary outlined the adult sample characteristics, study design/methods, MHLSC measures used and study outcomes allowing a high-level synthesis of the reviewed literature. For step 3, the data were then synthesized across different parameters in sequence; first studies were grouped according to sample (teacher, parent or mixed) to examine MHLSC by adult population. Next, studies were grouped by childhood MHP (ADHD vs others), then by MHLSC sub-component (e.g. knowledge and recognition and other sub-components), and then any covariates (such as age or gender of parent) of knowledge or recognition. For the final step, the strength of the synthesis was established by critiquing the quality of included papers.

## Results

### Search Results

**Fig. 2:**
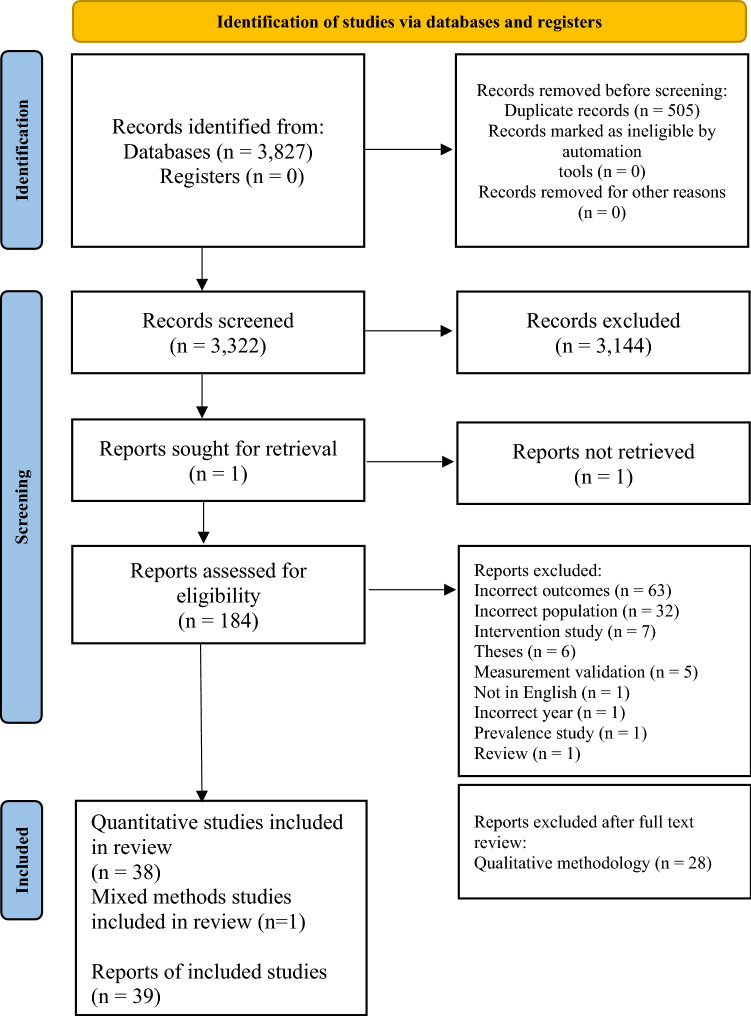
PRISMA diagram

Searches yielded a total of 3827 studies. After duplicates were removed, 3322 papers were screened and 184 underwent full text review, with 39 found to meet inclusion criteria. This process is summarized in Fig. 2. The main reasons for exclusion were studies not having at least one outcome related to knowledge or recognition of child MHPs, mixed child/adolescent mental health populations, or studies reporting on interventions.

### Characteristics of Studies

The included studies reported on teachers/school staff (*n* = 34), parents (*n* = 4), and mixed parent/teacher samples (*n* = 1). The samples tended to be majority female. The mean age of children with MHPs being examined across the studies could not be determined as not all studies reported on the exact age of the children (for example, reporting children by their grade level in school). Most studies that considered knowledge and recognition outcomes also measured other sub-components of MHLSC, the most commonly occurring being *Attitudes that facilitate recognition and help-seeking.*

Most studies (*n* = 27) were conducted in high-income Westernized countries, such as the United States (*n* = 7), the United Kingdom (*n* = 6), Australia (*n* = 6), Europe (*n* = 4), Ireland (*n* = 2) and Canada (*n* = 2). There were fewer studies (*n* = 12) conducted in low- and middle-income countries (LMICs). Most of the studies (*n* = 19) reported on teacher knowledge or recognition of ADHD. The remainder reported on parents’ or teachers’ general mental health knowledge across childhood disorders (*n* = 10), diagnosis-specific knowledge and recognition of internalizing disorders (*n* = 5), autism spectrum disorders (ASD) (*n* = 3) and both internalizing and externalizing disorders together (*n* = 2). Study characteristics are summarized in Table 1. A list of example outcomes that were coded under each sub-component is found in Table 2. More detailed study characteristics can be found in Supplementary File C.

**Table 1:** Characteristics of included studies

Study, country	Sample size & characteristics	MHP focus	Aim	Instruments	Relevant MHL outcomes	Quality assessment
*Teacher samples*						
Agyapong et al. (2010) Ireland	343 In-service	ASD	To examine the perception of primary school teachers about the recognition and management of Asperger’s syndrome	Author-developed survey—knowledge of symptoms of ASD	1,2	Low
Al-Omari et al. (2015) Jordan	130 *96.4% female*	ADHD	To examine the knowledge and attitudes of primary school teachers about ADHD	Self-report questionnaire based on Ghanizadeh et al. (2006)	1,2	Low
Al-Sharbati et al. (2012) Oman	263	ADHD	To describe Omani teachers’ awareness about ADHD	Self-report questionnaire based on Jerome et al., (1994), Oim (2004)	1	Low
Anderson et al. (2012) Australia	127 in-service 327 pre-service *n(female)* = *360*	ADHD	To test if teachers with different levels of general teaching experience differ in their knowledge of ADHD and their attitudes towards teaching children with ADHD	Perceived knowledge survey based on Smith et al. (2008), Knowledge About Attention-Deficit Disorder Questionnaire (KADD-Q)—shortened (West et al. 2005)	1,2,Co	Low
Bekle (2004) Australia	30 in-service 40 pre-service *n(female)* = *55*	ADHD	To compare the knowledge and attitudes of practicing teachers regarding ADHD to those of undergraduate education students	Questionnaire based on Jerome et al. (1994, 1999)	1,2,Co	Moderate
Bella et al. (2011) Nigeria	103 *n(female)* = *88* In- service	General	To determine the knowledge and attitudes of Nigerian elementary school teachers with regard to CAMH issues in schools	Authors-developed survey—piloted with 10 undergrads. 0.72 TRR reported	1,2,4	Low
Blotnicky-Gallant et al. (2015) Canada	113 *n(female)* = *99*	ADHD	To explore Nova Scotia teachers’ knowledge and beliefs about ADHD	Knowledge of Attention-Deficit Disorders Scale (KADDS), Beliefs About ADHD (B-ADHD) (Kos, 2008)	1,2	Moderate
Frigerio et al. (2014) Italy	579 *n(female)* = *565*	ADHD	To examine primary school teachers’ knowledge and perceptions of ADHD and analyse the relationships between perceptions, knowledge, and the teachers’ characteristics	Self-report questionnaire based on Norvilitis and Fang (2005) and ADHD Knowledge Scale (Jerome et al., 1994)	1,2,Co	Moderate
Ghanizadeh et al. (2006) Iran	196 *55.1% female*	ADHD	To survey the knowledge and attitude of elementary school teachers towards ADHD	Self-report questionnaire based on Brook et al. (2000) and Bussing et al. (1998)	1,2,4,Co	Low
Gowers et al. (2004) UK	186 In-service	General	To investigate teacher’s familiarity with mental health issues and experience with CAMHS	Authors-developed survey	1,2	Low
Groenewald et al. (2009) UK	212 *89% female*	ADHD	To investigate whether the type of ADHD symptoms influences teacher recognition of and views about referral and treatment for girls with ADHD	Author-developed surveys	1,2,3	Moderate
Headley and Campbell (2011) Australia	299 *n(female)* = *234* In-service	Internalizing (Anxiety)	To investigate the ability of teachers to identify anxiety problems of differing severity in children and their decision to refer to mental health services	Authors-developed Teachers’ Anxiety Identification and Referral Questionnaire (TAIRQ) and vignettes	1,2,Co	Low
Hutton et al. (2016) South Africa	51 In-service	ASD	To investigate what educators, know about ASD	The isiZulu KCAHW (Bakare et al., 2008)	1, Co	Moderate
Kerebih et al. (2016) Ethiopia	568 *n(female)* = *233* In-service	General	To assess the perception of primary school teachers towards school children’s mental health problems and their attitude towards school-based mental health services in southwest Ethiopia	Authors-developed survey, some questions drawn from Strengths and Difficulties Questionnaire	1,2,4,Co	High
Kleftaras and Didaskalou (2006) Greece	35 teachers *n(females)* = *19* In-service	Internalizing (Depression)	To estimate the proportion of primary school age children in one area of Greece displaying depressive symptoms and to examine teachers’ readiness and ability to identify and report those pupils	Students: Children Depression Inventory (CDI) (Kovacs, 1985) Teachers: Author-developed survey	1,4,Co	Low
Kos et al. (2004) Australia	165 *n(female)* = *91* In-service and pre-service	ADHD	To investigate the relationships between teacher characteristics and knowledge about ADHD, and to compare perceived and actual ADHD knowledge across in-service and pre-service teachers	Self-report questionnaire based on Jerome et al. (1994) and Sciutto et al. (2000)	1,Co	Low
Kypriotaki and Manolitsis (2010) Greece	365	ADHD	To examine the validity of teacher assessment in detecting children with ADHD the factors that influence teachers’ evaluations	ADHD rating scale-IV (DuPaul et al., 1998)	1,Co	Moderate
Layne et al. (2006) USA	Number of teachers not recorded In- service	Internalizing (Anxiety)	To establish whether teachers are aware of anxiety symptoms in their students using a nomination procedure and whether nomination is moderated by child age and/or gender	Students: Multidimensional Anxiety Scale for Children (MASC) Teachers: Author-developed survey	1,Co	High
Lee et al. (2015) Germany	234 *n(female)* = *204* Pre-service	ADHD	To investigate German pre-service teachers’ knowledge of ADHD & compare general and special education students	Knowledge of ADHD survey (Kos et al., 2004)	1,Co	Moderate
Loades and Mastroyannopoulou (2010) UK	113 *n(females)* = *82* In-service	Externalizing (ODD), Internalizing (SAD)	To investigate whether teachers can distinguish between children presenting symptoms of the same disorder at different levels of severity, their level of concern and factors predicting accurate recognition	Vignettes (ODD and SAD) based on two existing measures (Day, 2002; Stein et al., 2001), author-developed survey	1,2,Co	Moderate
Miranda Padilla et al. (2018) Colombia	62 *n(female)* = *61*	ADHD	To describe the knowledge of ADHD among primary school teachers in public schools	Knowledge of Attention-Deficit Disorders Scale (KADDS)—Spanish version	1,2	Moderate
Moldavsky et al. (2013) UK	496 *85% female*	ADHD	To investigate the relative effect of gender and subtype on teacher recognition of ADHD	Author-developed vignettes and survey	1,2,3,Co	High
Muanprasart et al. (2014) Thailand	201 *85.6% female*	ADHD	To provide a systematic assessment of knowledge regarding ADHD of Thai primary school teachers	Knowledge of Attention-Deficit Disorders Scale (KADDS)—Thai version	1,Co	Low
Neil and Smith (2017)* UK	51 In-service	Internalizing (Anxiety)	To assess how sensitive teachers were to variance in anxiety symptoms and teachers’ sensitivity to somatic symptoms in children	Students: Spence Children’s Anxiety Scale (SCAS), The Children’s Somatization Inventory (CSI) Teachers: Author-developed survey	1	Moderate
Ní Chorcora and Swords (2021) Ireland	356 *n(females)* = *296* In-service	Internalizing (Anxiety, Depression)	To explore teachers’ MHL and help-giving responses with regard to children presented with clinical and non-clinical levels of MHP’s	Author-developed survey with adapted questions from the Friend in Need Questionnaire (Burns & Rapee,^ 2006^) and vignette	1,2,Co	Low
Ohan et al., (2008a, b) Australia	140 *n(female)* = *119*	ADHD	To assess teachers’ knowledge of ADHD, explore how this knowledge is related to teachers’ reported behaviours towards and expectations of students with ADHD	ADHD Knowledge Scale (Jerome et al., 1994)	1,3,Co	Moderate
Sciutto et al. (2000) USA	149 *n(female)* = *134*	ADHD	To examine teachers’ knowledge and misperceptions of ADHD	Knowledge of Attention-Deficit Disorders Scale (KADDS)	1,Co	Low
Splett et al. (2019) USA	153 *n(females)* = *138* In-service	Internalizing, Externalizing	To examine variations in teacher-reported seriousness, concern, and need for intervention between externalizing and internalizing behaviour problems	Teacher Mental Health Literacy and Practices Survey (Loades & Mastroyannopoulou, 2010), vignette	1,2,Co	High
Topkin and Roman (2015) South Africa	200 *n(female)* = *178*	ADHD	To assess primary school teachers’ knowledge of the symptoms and treatment of ADHD;	Knowledge of Attention-Deficit Disorder Scale (KADDS), author created survey	1,4	Low
Vereb and DiPerna (2004) USA	47	ADHD	To explore the relationship between teachers’ knowledge of ADHD & knowledge of/preference for treatments for ADHD	Knowledge of ADHD Rating Evaluation (KARE) (developed by author –IC ranged from 0.58 to 0.81. TRR ranged from 0.76 to 0.80)	1,3,Co	Low
Walter et al. (2006) USA	254 *n(females)* = *119* In-service	General	To conduct a needs assessment survey to guide the development of a comprehensive program of mental health services	Author-developed survey	1,2,3	Moderate
Whitley and Gooderham (2016) Canada	186 (55% completed child version) Pre-service	General	To explore the mental health literacy of a sample of pre-service teachers	Author-developed survey, vignettes	1,2,Co	Moderate
Whitlock et al. (2020) UK	289 *n(female)* = *272* In- service and pre-service	ASD	To test if there is sufficient understanding among primary school teachers about autism in girls	Author-developed survey, vignettes	1,Co	Low
Woyessa et al. (2019) Ethiopia	206 *50.5% female*	ADHD	To investigate primary school teachers’ misconceptions about ADHD	Knowledge of Attention-Deficit Disorders Scale (KADDS)	1	Low
*Parent samples*						
Dang et al. (2021) Cambodia, Vietnam	357 mothers	General (SAD, ODD, SSD, Depression, Trauma)	To assess recognition of child mental health problems, beliefs about causes, parenting approaches, effectiveness of treatments for MHPs	Child Mental Health Literacy Questionnaire (CMHLQ, Shanley,^ 2009^), vignettes	1,4,Co	High
Huang et al. (2019) Australia	4983 children and their parents	General	To quantify under-recognition of children’s mental health problems by parents	Parent-reported Strengths and Difficulties Questionnaire (SDQ), parent report of current child MHP’s	1, Co	High
Lowinger (2009) USA	157 *n(mothers)* = *112* *n(fathers)* = *45*	General	To ascertain the extent to which parents would be willing to have their children referred for psychological help if they were manifesting emotional/behavioural problems in school and to identify factors that might affect parents' willingness to seek help	Author-developed survey, vignette adapted from Raviv et al. (2003)	1,2,3,Co	Moderate
Villatoro et al. (2018) USA	432 *M(age)* = 38.39 *n(Mothers)* = 341	General	To examine stigma's role in parental recognition of mental health problems	Parents: Author-developed survey, vignette Students: Author developed survey	1,2,Co	Moderate
*Mixed parent/teacher samples*						
Bevaart et al. (2012) Netherlands	17,511 *n(parents)* = *8,114* *n(teachers)* = *9,397*	General	To examine ethnic differences in problem perception and perceived need for professional care in parents and teachers of young children	Parents/teachers: Parent and teacher versions of SDQ	1,Co	High

Mental health literacy outcomes: 1—knowledge and recognition of mental disorders, 2—attitudes that facilitate recognition and help-seeking, 3—knowledge and beliefs about professional help, 4—knowledge and beliefs about self-help, 5—knowledge and beliefs about risk factors and causes, 6—knowledge of how to seek mental health information, *Co* study examined variables associated with knowledge/recognition

*ASD* autism spectrum disorder, *ODD* oppositional defiance disorder, *SAD* separation anxiety disorder, *GAD* generalized anxiety disorder, *ADHD* attention-deficit hyperactivity disorder, *BP* bipolar disorder, *SoAD* social anxiety disorder, *SSD* somatic symptom disorder, *MHP* mental health problem, *in-service* teacher in employment in a school setting, *pre-service* teacher still undergoing their teacher training

*Mixed-method study

**Table 2:** Examples of MHLSC outcomes from included studies grouped by MHL category

	Mental Health Literacy category	Examples of outcomes from included studies
1	Knowledge and Recognition of Mental Disorders	Recognition/identification of MHP Knowledge of MHP Knowledge of extent of MHPs (prevalence)
2	Attitudes that facilitate recognition and help-seeking	Attitudes towards MHP Stigma Confidence Perception of severity Level of concern Self-efficacy Intention to provide help
3	Knowledge and beliefs about professional help	Preference for seeking help/intervention type Understanding of treatment of disorder Knowledge of services available Beliefs about treatment/prognosis Self-rating of likelihood of referral to services
4	Knowledge and beliefs about risk factors and causes	Knowledge about what causes MHPs in children Attributes for MHPs in children
5	Knowledge and beliefs about self-help	Understanding of management of disorder Preference for and beliefs about classroom management of students (teacher only)
6	Knowledge of how to seek mental health information	NA

Ten studies employed a vignette survey design where teacher/parent recognition of diagnosis, knowledge of symptoms and appropriate help-seeking were measured through the presentation of a fictional child described as having symptoms of either a specific diagnosis or a general MHP (e.g. Loades & Mastroyannopoulou, 2010). Some studies compared adults’ responses across multiple vignettes that differed in severity (either mild, moderate or severe profiles described), child gender (some vignettes described the same MHP but varied in whether they depicted a female or male child) or MHP (for example, comparing recognition across vignettes depicting externalizing versus internalizing disorders). Five studies employed a screening design where a clinical screening tool was completed by the children or their parents (or the known mental health status of the children was used) to compare against teacher- or parent-rated child mental health status (e.g. Kleftaras & Didaskalou, 2006). Twenty-four studies conducted surveys, some using validated measures, asking teachers or parents to endorse or reject a range of statements about child mental health to examine knowledge (e.g. Agyapong et al., 2010).

### Results from Samples of Teachers

#### ADHD-Specific Studies

##### Primary MHLSC Outcome: Knowledge and Recognition of ADHD

Most papers (*n* = 19, 49%) reported on teacher knowledge of ADHD. Most of these employed a survey design, either as stand-alone questionnaires or in combination with vignettes. The two most commonly used validated measures were the Knowledge of Attention-Deficit Disorders Scale (KADDS) (Sciutto et al., 2000) which has three sub-scales (general awareness, symptoms/diagnosis and treatment) and the ADHD Knowledge Scale (Jerome et al., 1994). The percentage of correct answers on the KADDS ranged between 45 and 68.2%, and the ADHD Knowledge Scale between 69.5 and 82.9%. For studies that did not use standardized scales, scores ranged from 47.3 to 75.3%. Misconceptions, particularly about the causes of, treatment and prognosis of ADHD were particularly high in studies that were conducted in LMICs (Al-Omari et al., 2015; Al-Sharbati et al., 2012; Ghanizadeh et al., 2006).

Two studies used vignettes to assess teacher recognition of ADHD (Groenewald et al., 2009; Moldavsky et al., 2013). Both studies compared vignettes depicting a child with inattentive subtype ADHD with combined subtype ADHD. Recognition rates of ADHD were higher for the combined subtype of ADHD than the inattentive subtype—14% inattentive vs. 43% combined (Groenewald et al., 2009), 33% inattentive vs. 54% combined (Moldavsky et al., 2013).

Kypriotaki and Manolitsis (2010) used a screening design and found that only 9.1% of the boys and 74.3% of the girls attending Grades 1–3 who were initially nominated by their teachers as children with ADHD received scores above the cut-off point (85th percentile) on an ADHD identification scale (ADHD rating scale-IV (DuPaul et al., 1998). At the Grade 4–6 level, this rose to 88.4% of girls and 62.5% of boys.

##### Variables Associated with Knowledge and Recognition

*Teaching Experience* Some studies found teaching experience, especially that related to teaching children with ADHD previously, was associated with higher scores on knowledge scales, most often measured by comparing pre-service teachers to in-service teachers (Anderson et al., 2012; Bekle, 2004; Kos et al., 2004).

*Training* Many papers reported no or weak associations between prior training and knowledge of ADHD, although these studies varied in whether they assessed previous specialized training or simply exposure to the topic of ADHD in university level pre-service teaching degrees (Al-Omari et al., 2015; Kos et al., 2004; Ohan et al., 2008a, b; Sciutto et al., 2000). Relatively fewer papers found specialized training was associated with higher knowledge (Frigerio et al., 2014; Vereb & DiPerna, 2004), as was personal experience with family members or friends with ADHD (Muanprasart et al., 2014).

##### Secondary MHLSC Outcome: Attitudes that Facilitate Recognition and Help-Seeking

*Attitudes Towards ADHD* Negative attitudes, such as agreeing with the statements “ADHD students have lower IQ than non-ADHD students” or that “ADHD can be caused by parental spoiling” were more commonly reported in studies conducted in LMICs than those in higher income countries (Al-Omari et al., 2015; Al-Sharbati et al., 2012; Ghanizadeh et al., 2006). Teacher training in ADHD appeared to significantly influence the development of positive attitudes (Bekle, 2004).

In-service teachers appeared to have less favourable affective attitudes towards teaching students with ADHD than pre-service teachers (Anderson et al., 2012). Additionally, teachers’ beliefs about ADHD (but not their knowledge) were correlated with the use of effective behavioural strategies, such that those teachers with more negative beliefs about ADHD were less likely to use evidence-based behavioural management strategies (Blotnicky-Gallant et al., 2015).

*Perceived Need for Referral* The likelihood of referring children with ADHD for further professional help was examined as an outcome of interest in some studies. Teachers who correctly recognized ADHD were 13% more likely to refer children for help than teachers who labelled a problem as ‘attentional difficulties’ (Groenewald et al., 2009). Teachers were also more likely to support the use of medication if the child displayed combined subtype ADHD vs. inattentive subtype (Groenewald et al., 2009; Moldavsky et al., 2013). Knowledge also interacted with likelihood of seeking help, whereby teachers with high knowledge were more likely than those with low knowledge to endorse the need for and the intention to seek professional assessment for a child suspected to have ADHD (Ohan et al., 2008a, b). Those with low knowledge were more likely to indicate they would handle the child’s problems without assistance.

##### Secondary MHLSC Outcome: Knowledge and Beliefs About Professional Help, Risk Factors and Causes

Teachers were generally supportive of using education interventions and classroom management tools to manage ADHD, though attitudes towards medication were mixed (Blotnicky-Gallant et al., 2015; Frigerio et al., 2014; Topkin & Roman, 2015).

#### Non-ADHD Studies

##### Primary MHLSC Outcome: Knowledge and Recognition

The few cross-sectional surveys of teachers found low to moderate levels of knowledge and recognition of MHPs (Bella et al., 2011; Kerebih et al., 2016; Walter et al., 2006). Two studies measured teachers’ knowledge of ASD, and found teachers were more knowledgeable about the social challenges of ASD than the aetiological or language features. (Agyapong et al., 2010; Hutton et al., 2016; Whitlock et al., 2020).

Of the studies that measure recognition using vignettes, findings suggested that teachers were generally quite accurate in recognizing a hypothetical child with a MHP. Ní Chorcora and Swords (2021) found that most teachers could correctly label both anxiety and depression in a vignette (88.2% and 71.3%), and that they were sensitive to distinguishing between clinical and non-clinical severity, although the authors commented that only 3.9% of teachers used the term ‘anxiety disorder’. Similar indications of teachers’ high sensitivity to clinical vs. non-clinical levels of separation anxiety disorder (SAD) and oppositional defiant disorder (ODD) were found by Loades and Mastroyannopoulou (2010) although results from Headley and Campbell (2011) suggested teachers did have difficulty distinguishing between a child with moderate versus severe presentations of an anxiety disorder. Splett et al. (2019) found teachers could accurately identify both externalizing and internalizing disorders when the symptoms depicted were in the severe range but were less accurate in recognizing disorders depicted with moderate levels of symptoms. Further, when MHPs were depicted as moderate, teachers were better at identifying externalizing than internalizing disorders, and when asked to rate vignettes for severity, teachers rated the ‘seriousness’ of externalizing disorders higher than internalizing disorders. This finding was replicated in the Loades and Mastroyannopoulou (2010) study. A greater proportion of pre-service teachers in a study by Whitley and Gooderham (2016) using vignettes were able to identify anxiety and ADHD (89.6% and 84.40%), than depression and ODD (38.50% and 47.9%), though the depression vignette was rated as the most concerning by the teachers.

In contrast to the vignette studies, research using screening measures to validate adult’s recognition of real rather than hypothetical children with MHPs indicated mixed results. Validated measures such as the Children’s Depression Inventory (CDI), Spence Children’s Anxiety Scale (SCAS), or the Multidimensional Anxiety Scale for Children (MASC) were used to examine teachers’ ability to recognize internalizing disorders in classroom settings. Layne et al. (2006) found teachers were generally accurate at distinguishing between anxious and non-anxious students, while Neil and Smith (2017) found that teachers struggled to make this distinction. One study indicated teachers’ accurate screen-positive recognition of depression in children was only 14.1% (Kleftaras & Didaskalou, 2006). In general, the studies reviewed suggested that teachers were more successful at identifying internalizing disorders when they had some sort of outwards behavioural or somatic component (e.g. headaches/stomach-aches, extreme withdrawal) (Neil & Smith, 2017), and the most difficult symptom for teachers to recognize was anxiety manifesting as compliant behaviour (such as harm avoidance) (Layne et al., 2006).

##### Variables Associated with Knowledge and Recognition

*Child Gender* Teachers’ ability to recognize possible MHPs varied by child gender in some studies. This was particularly true for disorders with notable gender differences in presentation (e.g. ODD and ASD). In two studies, teachers presented with stereotypically gender-congruent vignettes (male ASD, male ODD, female SAD) were more accurate in problem recognition than those presented with gender-incongruent versions (Loades & Mastroyannopoulou, 2010; Whitlock et al., 2020). However, two other studies found no difference between teacher recognition of anxiety based on student gender (Headley & Campbell, 2011; Layne et al., 2006).

*Severity of MHP* Teachers struggled to discriminate recognition of a child with moderate anxiety symptoms from a child with a severe anxiety disorder (Headley & Campbell, 2011).

*Personal Experience* Teacher personal experience with mental health, through knowing or working with a child with a MHP, was found to be correlated with correct identification of depression in children (Ní Chorcora & Swords, 2021), and sensitivity to recognition of ASD (Whitlock et al., 2020).

*Teaching Experience* Teaching experience in and of itself appeared to predict teachers’ knowledge in the ADHD-related studies, in the non-ADHD studies the directionality of this relationship was less clear; three studies found no correlation between years of experience and scores on knowledge of ASD (Hutton et al., 2016), or accuracy in recognition of MHP’s (Kerebih et al., 2016; Loades & Mastroyannopoulou, 2010), but another found teaching experience and training predicted greater knowledge of MHPs (Walter et al., 2006).

*Training Received* Many teachers reported that they had brief or inadequate amounts of training in child mental health, either through initial teacher education courses/qualification (ITE) or through ongoing professional development (Agyapong et al., 2010; Gowers et al., 2004; Ní Chorcora & Swords, 2021; Walter et al., 2006). Specific training appeared to influence teacher confidence, as one study found that teachers who had received training reported greater levels of confidence in identifying ASD (Agyapong et al., 2010). However, in another study, training was not significantly associated with increased recognition of ASD (Whitlock et al., 2020).

##### Secondary MHLSC outcome: Attitudes that Facilitate Recognition and Help-Seeking

*Perception of Problem Severity* Teachers’ perception of the severity of a MHP was found to influence their likelihood of recognition and willingness to seek help on behalf of the child. Judgement of severity seemed to rely on a range of factors, including whether the child had received a diagnosis from a health care professional, the type of symptoms present (behavioural vs. emotional), the number of symptoms present and the overall impact on functioning. Judgement of the problem as ‘severe’ was associated with greater referral of children to professional services.

*Level of Concern* Higher levels of concern among teachers about a child’s wellbeing were associated with teachers’ greater referral of children to professional services (Headley & Campbell, 2011; Splett et al., 2019). Two studies found female teachers showed greater concern for and perceived MHPs as more severe, than male teachers did (Kerebih et al., 2016; Ní Chorcora & Swords, 2021), although another LMIC study found being female was associated with less tolerant attitudes towards MHPs (Bella et al., 2011).

*Type of Disorder* Some studies found that teachers had more concern for and endorsed greater ratings of severity for students with externalizing behavioural problems than for internalizing problems (Loades & Mastroyannopoulou, 2010; Splett et al., 2019) and that teachers were less likely to refer moderate levels of internalizing behaviour problems to community-based help than school-based help (Splett et al., 2019). Studies of pre-service teachers indicated student teachers showed the most concern for a child with depression, but only when this was paired with academic concerns (Whitley & Gooderham, 2016). Ní Chorcora and Swords (2021) found that teachers would seek external help for a child with depression (e.g. referral to a doctor) more frequently than for a child with GAD.

Some studies suggested that female teachers were more likely than male teachers to seek help for their students, for example, by referring to student wellbeing services (Headley & Campbell, 2011; Ní Chorcora & Swords, 2021). More years of teaching experience was also associated with decreased willingness to help.

##### Secondary MHLSC Outcome: Knowledge and Beliefs About Causes

Three studies considered teachers’ perception of the causes of MHPs in children. These found that teachers used primarily *relational* and *family environment/parenting* attributions for MHPs (i.e. teachers endorsed the belief that MHPs in children are caused by dysfunction in parenting style, home environment and/or familial or social relationships) (Kerebih et al., 2016; Kleftaras & Didaskalou, 2006; Whitley & Gooderham, 2016). In one study, this attribution pattern was stronger in older teachers (Kleftaras & Didaskalou, 2006).

### Results from Samples of Parents

Four studies, which explored the MHLSC of parents of children aged 5–12 years, were included in the review, as well as one study which explored parents and teachers’ perceptions together. All studies measured recognition of general MHPs in children, as opposed to singular diagnoses.

#### Primary MHLSC Outcome: Knowledge and Recognition

Two studies compared parent recognition of MHPs in their own child with results from validated screening measures (e.g. SDQ) and found parents tended to under-recognize problems in their children, with correct recognition ranging from 15.5% (for age group 4–7 years old), 54.4% (for age group 8–11 years old) (Huang et al., 2019) to 63.1% (for age group 5–6 years old) (Bevaart et al., 2012). Villatoro et al. (2018), Dang et al. (2021) and Lowinger (2009) used vignettes to study recognition and found generally low levels of recognition. Parents in Dang et al. (2021) identified a range of MHPs from vignettes, with correct recognition ranging from 17% (ODD) to 35% (for Trauma-Related Mental Health Problems), In Villatoro et al.’s study (2018), 29.1% of parents in the total sample recognized a mental health problem in their preadolescent child.

#### Variables Associated with Knowledge and Recognition

As with the teacher sample, most of the studies measured variables that influenced parent recognition. Villatoro et al. (2018) compared parent identification rates by the number of symptoms in their child by using the parent report DISC-IV screening tool to categorize children into a high-symptom and a low-symptom group. Parents were more likely to identify their child as having a MHP when their child was in the high rather than low-symptom group (57.7% vs. 12% accurate recognition). This study also used two child vignettes (a male with social anxiety and a female with bipolar). A greater proportion of parents identified the bipolar vignette as being indicative of a MHP (80.9%) than a social anxiety vignette (45.9%) (Villatoro et al., 2018). In line with this, Lowinger (2009) found that parents were more likely to perceive externalizing problems in a vignette as a problem, than internalizing problems, while Dang et al. (2021) found that internalizing disorders (such as depression) were recognized by mothers more readily than ODD, although recognition rates were low overall.

Parent characteristics, including family history of mental illness, lower desire for social distance from those with a MHP, familiarity with mental illness, female gender and higher family income, were also found to be associated with increased recognition (Villatoro et al., 2018), along with being part of an ethnic majority group (Bevaart et al., 2012). The finding of parents with higher income being more likely to recognize a MHP in their child was replicated by Huang et al. (2019). Their study also found that problem recognition was more accurate in parents of older children (8–11 years) than younger children (4–7 years).

#### Secondary MHLSC Outcome: Knowledge and Beliefs About Professional Help

Parents’ perceptions of their child’s need for professional help did not match their level of accurate recognition; although 63.1% of parents in Bevaart et al. (2012) study identified SDQ screen-positive children as having a MHP, only 22.9% reported a perceived need for mental health care. Results from Lowinger (2009) indicated that parents were more willing for a child with an externalizing disorder than internalizing to be referred to a school psychologist and agreed more often that help should be sought immediately for externalizing than internalizing disorders (87.6% vs. 78.9%). Villatoro et al. (2018) found that the more seriously a parent rated a problem, the more likely they were to endorse seeking help for it.

### Comparisons Between Teachers and Parents

Only one study consisted of both teacher and parent samples (Bevaart et al., 2012). The authors found that a higher proportion of teachers recognized and endorsed help-seeking for emotional and behavioural difficulties in children (as measured by the SDQ) than parents (87.2% vs. 63.1% for recognition and 47.8% vs. 22.9% for endorsing help in teachers vs parents, respectively). No statistical analyses were completed however, to determine whether the differences between parents and teachers were statistically significant.

### Quality Assessment of Studies

The outcomes of the AXIS quality assessment, for each of the included studies, are outlined in Table 1. The quality was most often low to moderate; only 7/39 (18%) were assessed as high quality. Most of the studies were limited by non-representative and non-rigorous sampling strategies, the use of non-validated measures and a lack of attention to non-respondents.

## Discussion

The aim of this review was to systematically examine the available quantitative evidence on the level of knowledge and recognition of MHPs of childhood among teachers and parents, as a sub-component of broader MHLSC. Our aim was to provide an overview of gatekeepers’ knowledge and recognition of childhood MHPs, and to identify potential targets for improvements as an avenue for increasing early intervention. The review found that studies of teachers’ knowledge and recognition of ADHD predominated, there was strong heterogeneity in measurement of adults’ knowledge and recognition of MHPs, there were few robust study designs with large sample sizes and a dearth of parent samples.

### Primary MHLSC Outcome: Parents’ and Teachers’ Knowledge and Recognition of Child MHPs

#### Knowledge

One area of MHLSC research was found to predominate the literature: teacher knowledge of ADHD. Teachers’ knowledge varied widely with a tendency towards scores in the middle of measures. When measured using standardized scales, teachers generally scored highest on sub-scales measuring knowledge of the symptoms/diagnosis of ADHD. Given that teachers commonly report teaching students with ADHD in the classroom, that ADHD has clear and quantifiable impacts on learning and that aspects of behaviour have the potential to impact classroom management, this is not a surprising finding. Other reviews, such as Mohr-Jensen et al. (2019), report similarly broad ranges of knowledge scores, with a tendency towards the upper range, especially on knowledge of disruptive symptoms like distractibility, hyperactivity and impulsivity.

Of the studies that surveyed groups of teachers and focussed on knowledge that did not pertain to ADHD, it is difficult to make conclusions because the results varied widely depending on the content of the survey questions (many of which were author designed) and the types of MHPs considered. There was a lack of consistent or validated measures used, and none that measured trans-diagnostic knowledge of childhood MHPs. However, the majority of these studies did not suggest very high or complete levels of knowledge of childhood MHPs.

It is surprising that there were no studies identified in this review that assessed parent knowledge of MHPs in children. Given that evidence exists to suggest that appropriate help-seeking can be facilitated by parental knowledge, and there are likely low levels of knowledge among the general population (as suggested by studies like the US National Stigma Study–Children), this may be an area for future researchers to focus on (Pescosolido et al., 2008).

#### Recognition

Significantly more studies reported on teacher and parent *recognition* of MHPs in children, particularly studies that considered MHPs other than ADHD. While teacher recognition tended to be generally adequate, parent recognition of MHPs in children was low across the four studies included. Notable differences were also found to exist between studies measuring recognition of MHPs via vignettes (hypothetical scenarios) and those that used real children whom adults lived or worked with. The evidence from vignette studies suggests that both teachers and parents were able to successfully recognize some MHPs (such as ODD and separation anxiety) and were able to critically select between clinical and non-clinical MHPs, especially for externalizing MHPs. In contrast, the studies that considered teachers’ identification of children they already worked with or knew, via screening the children with validated measures, demonstrated much lower accuracy in the teachers’ ability to identify MHPs. Similarly, parents’ ability to recognize MHPs was more accurate in vignettes than when asked to identify problems in their own children (Villatoro et al., 2018).

This finding may be the result of vignettes not accurately measuring adult knowledge and recognition of children with MHPs in real life situations, especially when parents or teachers may be faced with more complex comorbidities and subtle presentations that do not emerge in children they live or work with as a checklist of diagnostic symptoms, as is often portrayed in vignettes (Headley & Campbell, 2011). Furthermore, the impact of ecological factors, such as affiliate stigma, are likely to be important for adults of children when making decisions about when a MHP is present or absent (Mak & Cheung, 2008). Although vignettes are widely used in mental health research, some researchers have commented on their limitations, especially because the way adults react to and interpret a child’s behaviour in a real situation often differs significantly from the same behaviour presented in vignettes. For example, Lucas et al. (2008) asked teachers about their attributions, sympathy and help-seeking intentions towards a vignette and a real child in their classroom with “complex behaviours” arising from intellectual disabilities. In response to the vignettes, teachers were found to have stronger ratings of sympathy and help-giving intentions, than towards the real child. Staff were also more likely to attribute aggressive behaviours as within the child’s control when faced with a real child, than in the vignette (Lucas et al., 2008). Therefore, idealized scenarios with clear-cut clinical diagnoses, as presented in the vignette studies reviewed, may lack ecological validity for children. Future research is required to better understand how methods of measuring MHLSC impacts on results, especially with parents, as this was a particular gap found in the studies reviewed.

#### Variables Associated with Knowledge and Recognition

A complex range of variables were found to be associated with adults’ knowledge and recognition in this review. Adults generally found it easier to recognize certain MHPs when the child’s gender matched the stereotypical gender expected for diagnoses, such as male ASD/ODD/ADHD and female-anxiety (Kelter & Pope, 2011; Loades & Mastroyannopoulou, 2010; Molins & Clopton, 2002; Splett et al., 2019), and when children were older rather than younger (Huang et al., 2019; Kleftaras & Didaskalou, 2006). This may be due to a lack of confidence in identifying MHPs in younger children and a gender bias where adults expect externalizing disorders to be present in boys more so than girls. It could also be reflective of lower literacy for female presentations of MHPs, which may be associated with fewer disruptive behaviours (Molins & Clopton, 2002). This could, in part, explain the finding that teachers were more accurate in recognizing ADHD in girls simply because they had less false positives and were much more likely to overstate the prevalence in boys (Kypriotaki & Manolitsis, 2010). Training for gatekeepers likely needs to focus on a wider range of childhood MHPs beyond ADHD which was predominant in the literature covered. Training need also include patterns of MHPs that are not as strongly stereotyped, such as the presentation of ASD in females, and presentation patterns in younger children (Whitlock et al., 2020).

Having personal experience with MHPs, and having had training (specifically high quality, specialized training) was also associated with greater knowledge and recognition of MHPs. This finding is consistent with the wider literature on adult MHL, which has also reported the reinforcing effect on MHL of personal or family experience with MHPs (Hurley et al., 2020) and suggests knowledge and recognition of child MHPs can be improved with training and exposure in adults.

Interestingly, teachers who were more experienced in the classroom were less likely to recognize MHPs in children (except for ADHD) than early career teachers (Kerebih et al., 2016). Older teachers were also less likely to report favourable attitudes towards children with MHPs (Anderson et al., 2012) and more likely to attribute depression to family upbringing or parenting style (Kleftaras & Didaskalou, 2006). These findings perhaps reflect more recent trends in stigma reduction in wider public discourse (Pescosolido et al., 2021; Reavley & Jorm, 2012b) and suggests that interventions to improve teacher MHLSC may need to target stigmatizing attitudes in experienced teachers, as pre-service mental health training alone will be inadequate in upskilling the workforce.

Our review suggests that female adults and parents in higher income brackets are more likely to recognize and refer children with MHPs than male and adults with lower income were (Headley & Campbell, 2011; Ní Chorcora & Swords, 2021). This may be because the threshold of concern for mothers and female teachers may be lower than for males, although arguably it could be reflective of a gender bias towards surveying females in parenting and education studies. Recent international studies have indicated that females have higher overall MHL (Hadjimina & Furnham, 2017; Mendenhall & Frauenholtz, 2013; Pescosolido et al., 2008) and more positive help-seeking intentions (Yu et al., 2015), and generally men are less likely to endorse seeking help for MHPs. These attitudes may also likely be applicable to male teachers’ and parents’ MHLSC and their help-seeking for children (Oliver et al., 2005). From this review, there also appears to be little research on how social inequities and intersecting marginalized identities might impact on knowledge and recognition of MHPs in children. In the broader MHL literature, research has shown that there are important differences in MHL among adults from different racial and cultural backgrounds, but to date, little research has focussed on class, inequity or intersectionality (Loo et al., 2012).

### Secondary MHLSC Outcome: Attitudes that Affect Recognition and Help-Seeking

This review found a range of attitudes that appear to affect recognition and help-seeking, including adults’ reported level of concern, their confidence in seeking help, their perception of the severity of a problem and their perception about the type of MHP. Generally, among the studies reviewed, it was found that if an adult perceived a problem to be severe, had positive beliefs about MHPs, had concern for a child and felt confident in their ability to support a child, then they were more likely to seek help and to use effective strategies to support children. This finding is consistent with previous research which has shown that the more serious or severe an adult perceives a child’s problem to be, the more likely they are to conceptualize it as a MHP (rather than the more vague ‘behavioural issue’), and to initiate help-seeking, and more specifically, contact with mental health services (Ford et al., 2008; Groenewald et al., 2009). Further, it seems likely that attitudes are an important component of MHLSC given they were frequently reported on in conjunction with knowledge and recognition.

Mixed evidence was found for differences across how parents and teachers perceive and respond to internalizing versus externalizing MHPs. Although some studies showed adults held greater concern for and rated externalizing disorders as more severe, this was not a consistent finding. This is in contrast to findings from other research that suggests externalizing disorders are often recognized more readily, are perceived as more of a concern and that service-seeking for externalizing disorders is more common (Molins & Clopton, 2002; Pihlakoski et al., 2004; Sayal, 2006; Thurston et al., 2015). Further research is needed to determine if adults under-recognize internalizing MHPs in children, or whether they perceive internalizing to be less serious than externalizing MHPs, and whether these issues could be addressed in population level training interventions.

### Secondary MHLSC Outcome: Beliefs About Causes

In the studies reviewed, it was reported that adults tended to assess MHPs as caused by either biological factors or the family/parental environment of the child. In previous research causal beliefs regarding the etiology of MHPs have been linked to adult behaviour and decision making, and importantly to positive treatment outcomes and teacher management (Hoza et al., 2000; Mikami et al., 2019; Sawrikar et al., 2018, 2020). Adult causal beliefs that are recovery orientated and highlight the amenability of MHPs to treatment are likely to result in better outcomes for children (Chaidez et al., 2018; Yeh et al., 2005).

### Reliability of Studies and Limitations

Although this review was able to provide an overview of the available evidence on MHLSC among parents and teachers, there were some limitations. All of the studies included in this review were observational and cross sectional, which limited the ability to establish comparisons across groups, and most were rated using AXIS as low or moderate quality. Many studies did not sample the population of interest with representative sampling frames. A significant limitation of the included studies is that there was no consistent measure of MHLSC used, with many studies using author-developed tools that did not report psychometric properties. This highlights a major shortcoming in the existing research. Similarly, although vignettes were largely based on previous studies or MHPs defined by the Diagnostic and Statistical Manual of Mental Disorders (American Psychiatric Association, 2013), there was little consistency across studies, even among vignettes designed to measure knowledge of the same disorder. Furthermore, as previously argued, adults may not respond in the same way to a hypothetical vignette as they would to their own child that they live with or teach, so findings arising from vignette studies may be further limited in their validity.

Intervention studies were excluded from the review, so it is possible that relevant results, which support or contradict the findings presented here, have not been included. Although the justification for the narrow definition of MHLSC used in the current review was well supported by other researchers calling for more stringent conceptualization (Spiker & Hammer, 2019), there were papers that were rejected from this review because they covered adjacent areas of interest (e.g. the impact of stigma on recognition) but did not report directly on knowledge or recognition; the primary inclusion criteria. Qualitative results were also excluded from the current review, given the divergent outcomes from different methods which could not easily be compared with the studies in this review.

### Implications of Findings and Future Research

Some aspects of the reviewed literature were difficult to synthesize because of disparate definitions, methods, measures and outcomes. This difficulty could be overcome in future research by establishing a core definition and conceptual scope for MHLSC, and how it differs from adult or adolescent MHL. We suggest that these foundational elements are pressing needs for future research as they would aid the field in moving on to broader questions—like how MHLSC can be improved and what are the benefits of doing so—by allowing the synthesis of findings across methods, measures and populations. Another gap established in this review was the lack of population level surveys for measuring adults’ MHLSC. Effective measurement of population MHLSC over time, such as the nationally representative surveys tracking adult and adolescent MHL over several years and even decades (Reavley & Jorm, 2012a), could be an important innovation in this field as it could uncover novel targets for MHLSC interventions (Tully et al., 2019) and inform national policy. However, such large-scale research requires standardized, robust and valid MHLSC measures to be used.

Furthermore, the conceptualization of mental health in children needs further attention. In Australia, the new National Children’s Mental Health and Wellbeing Strategy 2021 (National Mental Health Commission, 2021) recommends use of a mental health continuum based on levels of functional impairment and distress, rather than on diagnostic classifications or focussing on the internalizing/externalizing divide. This approach better aligns with the dimensional Hierarchical Taxonomy of Psychopathology (HiTOP) empirical classification and shows better validity in psychiatric research than many of the DSM delineations (Kotov et al., 2017; Ruggero et al., 2019). We believe that encouraging parents and teachers to both use a consistent continuum framework that focusses on functioning and distress (rather than symptom categories) would aid better assessment and earlier intervention.

Both parents and teachers are primary informants for health care professionals and have a central role in early identification and help-seeking (De Los Reyes et al., 2015). To improve early intervention in childhood, future research in this area could close important gaps in the current literature. There has been far less measurement of parents’ MHLSC than teachers, although this is possibly covered by literature with qualitative approaches or indicated studies with parents who already have children with a diagnosis. However, understanding how robust parents’ MHLSC is before their children are at the point of receiving a diagnosis, and what level of MHLSC is sufficient or adequate to prompt help-seeking for children, are imperative in designing early intervention approaches. This is a key area for future research given the centrality of parents in the help-seeking process for children, and preliminary evidence indicating links between high MHL and positive help-seeking intentions in other parent populations (Cormier et al., 2020). To this end, we are conducting a separate qualitative synthesis to better understand how attitudes or beliefs may influence knowledge and help-seeking behaviours among parents and teachers (Gross et al., 2020). There may be other modifiable factors that could be potential targets for intervention design for parents and teachers. Future research could also systematically review what interventions already exist, and what level of evidence they have for improving the MHLSC of parents and teachers, with the goal of identifying what programs or elements might be most effective for targeting these populations.

Finally, in opposition to the large literature on ADHD, there was a notable lack of studies exploring recognition and knowledge of other important childhood disorders, such as eating disorders or OCD. Further investigating how well parents and teachers understand and can recognize these disorders should also be targets for future studies.

## Conclusion

This review explored the MHLSC among parents and teachers, with a specific focus on the sub-components of knowledge and recognition. This review found that although parents and teachers are well placed to identify childhood MHPs, there are aspects of their knowledge and recognition of MHPs that are poorly developed and could be the target of future research and interventions. Furthermore, important gaps in the current research were noted, especially in the use of standardized measures of MHLSC, and in our understanding of adults’ knowledge and recognition of child MHPs outside of ADHD. Nevertheless, the findings from this review justify a specific focus on MHLSC training for parents and teachers and highlights the need for more targeted research in this area, to achieve better outcomes in child mental health.

## Supplementary Information

Below is the link to the electronic supplementary material.

Supplementary File A: Inclusion and Exclusion Criteria (DOCX 14 KB)

Supplementary File B: Search Terms and Mapping (DOCX 20 KB)

Supplementary File C: Table of Characteristics of Included Studies (DOCX 90 KB)

## Data Availability

Supplementary material, such as data coding templates and assessment of risk of bias forms can be requested from the first author using the contact details provided.
