# Supplementary material for: Mental Health Literacy for Supporting Children: A Systematic Review of Teacher and Parent/Carer Knowledge and Recognition of Mental Health Problems in Childhood
Source: Clin Child Fam Psychol Rev. 2023 Feb 10;26(2):569–91. doi: 10.1007/s10567-023-00426-7 (PMC10123050; doi:10.1007/s10567-023-00426-7)
Supplement: Supplementary file 1 — Supplementary File A: Inclusion and Exclusion Criteria (DOCX 14 KB) [file 10567_2023_426_MOESM1_ESM.docx]

**Supplementary File A**

*Inclusion and Exclusion Criteria*

| Inclusion criteria | Exclusion criteria |
| --- | --- |
| Published in English | Published in language other than English |
| Published in the year 2000 -2021 | Published prior to 2000 |
| Peer reviewed articles | Grey literature or theses |
| Quantitative studies, mixed methods studies with quantitative results | Qualitative studies |
| Measurement of MHL in adult primary school teachers, biological parents, foster parents, or any other caregivers of children | Measurement of MHL in children or adolescents |
| Age of the population with the mental health problem between 5 and 12 years old | Age of population of children is <5 years or >12 years, or age is not reported |
| Population aged mixed (e.g. children aged 4-17 years old) results delineated by age sub-groups | Population of children is mixed and results are not reported by sub-group |
| Papers addressing both MHL with regards to specific disorders (e.g. autism spectrum disorder, anxiety) and general mental health problems | Papers addressing problematic child behaviours, (e.g. disruptive behaviour, lack of concentration) |
|  | Papers focusing on indicated populations, such as teacher or parent ongoing management of existing mental health problems |
| Measures at least one outcome to do with recognition or knowledge of mental health problems in children |  |
|  | ‘Measurement’ studies that validate an existing scale or seek to explore a new instrument of mental health literacy. |
|  | Intervention studies |
