# Supplementary material for: Mental Health Literacy for Supporting Children: A Systematic Review of Teacher and Parent/Carer Knowledge and Recognition of Mental Health Problems in Childhood
Source: Clin Child Fam Psychol Rev. 2023 Feb 10;26(2):569–91. doi: 10.1007/s10567-023-00426-7 (PMC10123050; doi:10.1007/s10567-023-00426-7)
Supplement: Supplementary file 2 — Supplementary File B: Search Terms and Mapping (DOCX 20 KB) [file 10567_2023_426_MOESM2_ESM.docx]

**Supplementary File B**

*Search Terms And Mapping*

**1.** (“Mental health literacy” OR “literacy” OR “perception*” OR “competenc*” OR “understand*” OR “abilit*” OR “capability*” OR “confiden*” OR “know*” OR “identif*” OR “aware*” OR “recogni*” OR “label*”)

AND

**2.** (“Mental health OR “mental illness” OR “mental disorder” OR “depress*” OR “anxi*” OR “mood disorder” OR “affective disorder” OR “disruptive behave*” OR “eating disorder” OR “internali*” OR “externali*”)

AND

**3.** (teacher* OR Parent* OR Mother OR Father OR Famil* OR Caregiv*; OR guardian* OR “gatekeeper*” or “gate-keeper*” or “gate-keeper*”)

AND

**4.** (primary or junior or elementary or grade) AND school* OR child* OR student*

| Search Term | APA Index (PsychINFO) | MeSH heading (MEDLINE) | Thesaurus search (ERIC) |
| --- | --- | --- | --- |
| Literacy | | | |
| Literacy | Mental Health Literacy | Health Literacy | N/A |
| Perception | Perception | N/A | [Perception](about:blank) |
| Competence | Competence |  | [Competence](about:blank) |
| Understanding | Comprehension | Comprehension | [Comprehension](about:blank) |
| Ability | Ability | Aptitude | Ability |
| Confidence | Confidence | Confidence | Confidence |
| Knowledge | Health Knowledge  Knowledge (General) | Knowledge | NA |
| Identification | Identification |  | Identification |
| Awareness | Awareness | Awareness | [Perception](about:blank) |
| Recognition | Recognition (Learning) | Recognition, Psychology | Recognition (psychology) |
| Label | Labelling |  | Labelling (of persons) |
| Mental illness | | | |
| Mental health | Mental Health | Mental Health | Mental Health |
| Mental illness | Mental disorders | Mental disorders | Mental disorders |
| Depression | Major depression  Depression (Emotion) | Depression  Depressive Disorders | Depression (psychology) |
| Anxiety | Anxiety  Anxiety Disorders  Generalized Anxiety Disorder  Separation Anxiety  Separation Anxiety Disorder  Social Anxiety | Anxiety  Anxiety Disorders  Anxiety, Separation  Phobia, Social | Anxiety  Anxiety disorders |
| Mood Disorder | Affective disorders | Mood disorders  Affective symptoms  Affective disorders | NA |
| Disruptive Behaviour | Behaviour Problems  Behaviour Disorders  Disruptive Behaviour Disorders  Classroom Behaviour  Aggressive Behaviour  Classroom Behaviour Modification  Classroom discipline | Disruptive Behavior Disorders  Conduct disorders  Antisocial Personality Disorder | Antisocial behavior  Behavior disorders  Behavior problems |
| Eating Disorder | Eating disorders |  | Eating disorders |
| Internalizing |  | No equivalent | NA |
| Externalizing | Attention Deficit Disorder with Hyperactivity  Conduct Disorder  Oppositional Defiant Disorder  Autism Spectrum Disorders | No equivalent | NA |
| Population | | | |
| Children | No equivalent term | Child  Child, preschool | Children |
| Student | Elementary school students  Middle school students  Primary school students | Students | Elementary schools  Elementary education |
| Teacher | Elementary school teachers  Middle school teachers  Teachers | School teachers  *Narrowers:* Elementary School Teachers  Pre-school Teachers | Elementary school teachers |
| Parent | Parents | Parents | Parents |
| Family | Family | Family | Family (sociological unit) |
| Mother | Mothers | Mothers (human and animal) | Mothers |
| Father | Fathers | Fathers (human and animal) | Fathers |
| Caregivers | Caregivers | Caregivers  *Narrowers:* Family caregivers | Caregivers |
| Guardian | Guardianship | Legal Guardians | NA |
| Gatekeeper | No equivalent term | Gatekeeping | NA |
