# Supplementary material for: Mental Health Literacy for Supporting Children: A Systematic Review of Teacher and Parent/Carer Knowledge and Recognition of Mental Health Problems in Childhood
Source: Clin Child Fam Psychol Rev. 2023 Feb 10;26(2):569–91. doi: 10.1007/s10567-023-00426-7 (PMC10123050; doi:10.1007/s10567-023-00426-7)
Supplement: Supplementary file 3 — Supplementary File C: Table of Characteristics of Included Studies (DOCX 90 KB) [file 10567_2023_426_MOESM3_ESM.docx]

**Supplementary File C**

*Table of Characteristics of Included Studies*

| Study Author (Year), Country  *Inservice or preservice teacher sample (where applicable)* | MHP Focus, Sample Size,  Age (years), Gender, Ethnicity, Teaching experience | Relevant Knowledge/Recognition outcomes | Other outcomes | Variables: knowledge/ recognition | Key Results |
| --- | --- | --- | --- | --- | --- |
| Teacher samples | | | | | |
| Agyapong et al. (2010)  Ireland  *In-service* | ASD  343  NR  NR  NR  58.9% of sample > 5 years | Recognition of specific ASD symptoms | Confidence in recognising ASD symptoms  Perception of whether ASD children should be taught in mainstream schools  Perception of what resources should be available to schools on ASD  Perception of how ASD should be managed  Training undertaken in undergraduate course for ASD | None | - Teachers responded to a survey asking them to rate their ability to recognize symptoms of ASD and nominate which symptoms indicated ASD - All teachers reported they had heard of ASD - 78.4% of teachers reported they believed they would recognise symptoms of ASD in a child. - 97% of teachers classified Asperger’s as an ‘autistic disorder’ whilst the remaining said it was a ‘psychiatric disorder’. - ASD symptom best recognised by teachers was "Intense absorption in certain subjects" (90.4%), lowest recognition: "abnormal, repetitive speech" (58%). |
| Al-Omari et al. (2015)  Jordan  *In-service* | ADHD  130  NR  96.4% female  NR  75.4% of sample > 5 years | Knowledge of ADHD | Attitude towards children with ADHD | Age  Marital status  Place of work  Educational level  Teaching experience.  Level of exposure to ADHD information | - Teachers completed a survey asked them to respond to statements about ADHD and indicate if they were true/false - The mean score for teachers’ knowledge on the ADHD scale was 6.9, (range=4-11), no one achieved the maximum score of 12. - False statements such as ADHD being linked to sugar and food additives were endorsed by 64.8% - The majority of teachers had a negative attitude towards children with ADHD. - No significant relationships found between teacher age, experience, marital status, place of work or level of exposure to ADHD information and knowledge or attitude towards ADHD |
| Al-Sharbati et al. (2012)  Oman  *In-service* | ADHD  263  NR  NR  NR  *M* = 9.2 years | Knowledge of ADHD | None | None | - Teachers completed a survey asked them to respond to statements about ADHD and indicate if they were true/false - 73% agreed with “ADHD is caused by poor parenting” - 72% agreed with “Child with ADHD has good school performance” - 55% agreed with “Heredity causes ADHD” |
| Anderson et al. (2012)  Australia | ADHD  127 in-service, 327 pre-service  *M* = 41.04 (in-service), *M* = 28.78 (pre-service with no teaching experience)  79.3% female  NR  *M* = 15.7 years | Knowledge of ADHD, perceived knowledge of ADHD | Attitude towards ADHD, attitude content  Personal experience with ADHD | Teaching experience (pre-service, pre-service with some experience, in-service) | - Teachers completed a survey that asked them to rate statements on ADHD causes, characteristics and treatments as true/false/don’t know - Three groups for comparison: pre-service teachers with teaching experience, pre-service teachers without teaching experience, in-service teachers - Total knowledge: Inservice: M= 19.88 (SD= 4.52), pre-service with experience: M= 17.45 (SD=5.38) pre-service without experience: M=17.21 (SD=5.41) - In-service teachers had significantly higher total knowledge of ADHD and higher perceived knowledge than did pre-service teachers (F(2,446) = 10.82, p< .001, ƞ2p = .05) - In-service teachers reported significantly less favourable affect than did pre-service teachers with no experience (F(2,330) = 7.01, p = .001, ƞ2p = .04.) |
| Bekle (2004)  Australia | ADHD  30 in-service, 40 pre-service  NR  78.57% female  NR  Majority of sample > 8 years | Knowledge of ADHD | Attitude towards ADHD  Training undertaken on ADHD | Teaching experience (in-service, pre-service) | - Teachers completed a survey that asked them about ADHD training received, amount of contact teachers had with ADHD students, and participants’ knowledge of basic concepts of ADHD - In-service teachers (M = 16.57, SD = 1.8) scored significantly higher than undergraduate education students (M = 15.03, SD = 1.9) on overall knowledge (out of 20) - In-service teachers appeared to be better informed than undergraduate students in the area of causation. The majority of teachers (83%) compared to only 43% of students agreed with the statement that “ADHD can be inherited.” - No attitude differences were detected between groups, although knowledge and attitude were positively correlated |
| Bella et al. (2011)  Nigeria  *In-service* | General  103  *M* = 41.5  85.44% female  NR  12.7 years | Knowledge of MHPs | Attitude towards MHPs | Gender of teacher  Religion  Location (urban vs. rural)  Educational Level | - Teachers responded to a survey asking them about awareness of common mental health problems in children, signs and symptoms of these problems, social distance towards children with mental health problems (willingness to interact with them), stigmatising terminology used for mental health problems and cultural attitudes to mental health problem - 5 of the 19 questions received over 70% correct agreement with a true statement - Slightly more than half of the respondents agreed with the statements that ‘epilepsy is a type of mental health problem’ (52.4%), ‘mental health problems in children are rare’ (51.5%), ‘moron is a term for learning disability in children’ (64%), ‘children who are restless and disturb the class are extroverts’ (57.3%) and ‘poor academic performance may be a sign of a mental health problem’ (53.4%) - There was very low agreement and recognition of signs of mental health problems in school refusal (23.3%), children not doing their homework (12.6%) and inability to pay attention in class (31.1%). - While most teachers would accept children with mental health problems in their classes, the majority felt they would be better off in special schools. Less tolerant attitudes were associated with lower educational qualifications and working in a rural setting. |
| Blotnicky-Gallant et al. (2015)  Canada  *In-service* | ADHD  113  *M* = 42.5  87.61% female  NR  *M* = 15.5 years | Knowledge of ADHD | Attitudes towards ADHD  Use of interventions for students with ADHD | None | - Teachers completed a survey asked them to respond to statements about ADHD and indicate if they were true/false - Total scores on the KADDS ranged from 25.7% correct to 91.4 % correct, with a mean of 68.2% correct (SD = 11.9) - Perceived knowledge was not correlated with actual knowledge - Attitudes: Highest negative beliefs found on the External Control subscale (M = 3.26, SD = 0.53) (e.g. indicating students with ADHD can be more difficult to teach) and the least negative beliefs on the Diagnostic Validity subscale (M = 1.79, SD = 0.60) (e.g. indicating teachers thought ADHD was a valid diagnosis) |
| Frigerio et al. (2014)  Italy | ADHD  579  *M* = 44.4  96% female  NR  *M* = 19.19 years | Knowledge of ADHD | Attitude towards ADHD | Teaching experience  Level of exposure to ADHD information | - Teachers completed a survey asking them about their ADHD knowledge and attitude towards ADHD - Mean score of ADHD Knowledge was 13.21 (SD=2.25) out of 19 - Attitudes: Teachers were negative toward the use of medication and tend to not rely on discipline as an effective tool in the management of ADHD. Agreed that ADHD is caused by a biological predisposition that can affect all stages of life, but that does not directly influence mental skills, like smartness or creativity. Perceived ADHD as underdiagnosed, but that existing social services are adequate - Knowledge positively correlated with some sub-domains of attitudes, including disagreement that disciple is the best method to handle students with ADHD, belief that ADHD is underdiagnosed and the affirmation of the long-term effects of ADHD - Teaching experience was negatively correlated with knowledge of ADHD |
| Ghanizadeh et al. (2006)  Iran | ADHD  196  *M* = 38.92  55.1% female  NR  *M* = 19.11 years | Knowledge of ADHD | Attitude towards ADHD | None | - Teachers completed a survey asked them to respond to questions regarding knowledge, attitude and source of acquired information on ADHD - Knowledge score 3.79 (SD=1.15) (out of 8) - Attitude score 7.42 (SD=2.33) (out of 12) - Knowledge weakly positive correlated with attitude and educational level - About 46% of respondents agreed that ADHD is due to biological and genetic vulnerabilities. However, more than half of them believed that it is due to parental spoiling. One-third of respondents thought that ADHD may be caused by excessive sugar intake - Moderate negative attitudes towards students with ADHD were found |
| Gowers et al. (2004)  United Kingdom  *In-service* | General  186  NR  NR  NR  64.52% of sample > 20 years | Knowledge of prevalence of MHPs | Difficulties faced in teaching children with MHPs  Confidence in understanding MHPs  Training undertaken on MHPs  Experience of CAMHS | None | - Teachers responded to a survey asking them about perceived prevelance of MHPs, perceived difficulties in teaching children with MHPs, confidence in understanding of MHPs, prior training in MHPs and experiences with CAMHS. - **Prevalence:** Majority of teachers reported they encountered MHPs quite frequently (41%) - **Confidence in understanding of MHPs:** Majority of teachers selected inadequate or fairly inadequate (24% + 32%) |
| Groenewald et al. (2009)  United Kingdom | ADHD  212  *M =* 41.3  89% female  NR  56% of sample > 10 years | Recognition of ADHD | Need for referral  Identification of preferred intervention | Sub-type of ADHD (inattentive vs. combined) | - Teachers responded to a vignette describing a 9-year old girl with either combined subtype (hyperactivity, impulsivity and inattention) or predominantly inattentive subtype ADHD - Recognition of ADHD: Majority of teachers indicated the vignette was indicative of ‘attentional’ or ‘emotional’ problems – only 14% of teachers identified ADHD (for inattentive subtype) and 43% (for combined subtype) - Need for referral: Likelihood of teachers considering a need for referral was 59% for combined subtype and 49% for inattentive subtype ADHD. Conceiving of the problem as ‘attentional difficulties’ reduced the teachers likelihood of indicating they would seek help by 13% (95% CI 5–21%; p = 0.002) - All the intervention approaches were strongly endorsed: learning support (98%), work with parents (94%) and behaviour therapy (89%). In contrast, only 15% of teachers thought that medication might be helpful. The type of vignette influenced their endorsement of medication (21% for combined and 9% for inattentive subtype vignettes; p = 0.025). |
| Headley and Campbell (2011)  Australia  *In-service* | Internalizing (Anxiety)  299  *M* = 40  78.3% female  NR  *M* = 16 years | Identification of anxiety | Decision to refer children for further help | Severity of disorder (minimal, mild, moderate, severe, very severe)  Gender of teacher  Gender of child | - Teachers responded to five vignettes with varying levels of severity (ranging from “Mild” to “Very Severe”) and answered questions on identifying children in need of referral, and ranking vignette children in terms of urgency for referral. - The effect of severity of anxiety on the identification (by way of need for referral) in the vignettes was significant, *F* (3.53, 847.78) = 257.83, p < .001, partial ƞ2 = 0.52.) The analysis indicated that teachers had difficulty distinguishing between a child with moderate anxiety symptoms and a child with a severe anxiety disorder. - Effect of severity of anxiety on the ranked urgency to refer a child in the vignettes was significant, (*F* (3.89, 1104.65) = 315.57, p < .001, partial ƞ2 = 0.53.) The analysis indicated that teachers decided to make referrals for students with moderate anxiety symptoms more than those with severe anxiety symptoms |
| Hutton et al. (2016)  South Africa  *In-service* | ASD  51  *M* = 46.3  NR  100% Black South African  *M* = 19.29 | Knowledge of ASD | None | Age  Teaching experience | - Teachers responded to a survey asking them about four domains of ASD; impairment in social interaction, impairment in communication, obsessive and repetitive behaviours and the nature of ASD and its co-morbidity. - Educators scored an average total score of 68% out of a possible 100%. The mean total score for the educator sample was M = 13.08/19 - Domain four (knowledge about the aetiology of ASD) was the most challenging for the educators. (M = 2.73/6) - Domain two (knowledge about impairment in communication) was least challenging (M = .78/1) - A correlation analysis did not reveal any significant correlations between the educators’ age, years of teaching experience and total scores. |
| Kerebih et al. (2016)  Ethiopia  *In-service* | General  515  *M* = 42.39  45.24% female  48.7% Oromo ethnic group  21.9% Amhara ethnic group  12.6% Yem ethnic group  6% Dawuro ethnic group  75.5% of sample > 9 years | Recognition of mental health problems | Perception of severity  Beliefs about causes | Gender of teacher  Age  Religion  Marital status  Level of education  Training in MHPs  Size of classes taught  Year level taught  Teaching experience | - Teachers responded to a survey with a list of symptoms asking them about whether those symptoms constituted a MHP, the severity of these problems and their attitude towards mental health service needs - **Recognition of MHPs:** More than 60% of teachers perceived symptoms (such as “Often unhappy, depressed or tearful”) as not being a mental health problem. Among the teachers who did perceived symptoms as MHPs, externalizing [i.e., hyperactivity (41.4%) and conduct problems (37.0%)] type symptoms were classified as MHPs more than internalizing [emotional problems (32.4%) and peer-related problems (35.3%)]. - **Beliefs about causes:** Most often identified cause was social problems (61.7%) followed by problems with family (54%) and poor school and home environment (52%). Genetics/heredity (31.5%), medical illness (30.9%), and spiritual/sin (15.3%) were rated as the least likely causes of child mental health problems by the teachers - In a multiple logistic regression analysis, less years of teaching experience and teaching in a government school was associated with greater likelihood of perceiving severe types of children’s mental health problems was 15 times more likely to classify symptoms as MHPs |
| Kleftaras and Didaskalou (2006)  Greece  *In-service* | Internalizing (Depression)  35 teachers, 323 students  NR  54.3% female (Teachers), 52.3% female (students)  NR  NR | Teacher perceptions of depression in classroom | Student levels of depression  Teacher perceptions of causes of depression | Age of teacher | - Students completed the CDI and teachers responded to a survey asking them about the number of pupils in their classroom who, according to their personal judgement, displayed problematic behaviour or behaviour that caused them concern and rate the frequency of occurrence of each item-behaviour on a weekly basis. Teachers were asked to number and describe the perceived causes of the problems that were being displayed by those particular students in the classroom whom they identified as exhibiting problematic behaviour or behaviour - 99 students reported mild and severe depressive symptoms on their CDI, however, teachers only identified 14 of these as having “emotional or behaviour problems” (14.1%). - Teachers identified 34/323 students as exhibiting emotional or behavioural problems. Among these 34 students, 20 reported no depressive symptoms, while only three reported severe depressive symptoms, and 14 reported mild depressive symptoms - **Beliefs about causes:** When teachers were asked to indicate the cause of student symptoms, teachers commonly reported problems within families, such as family dysfunction, adverse family background and inappropriate parenting skills. - In an ANOVA, younger (25-35) and older teachers (47+) but not those of the middle age group category were more likely to attribute students’ problems to ineffective parenting skills and indulgent rearing practices. |
| Kos et al. (2004)  Australia  *In-service and pre-service* | ADHD  165  *M* = 39.2  55.15% female  NR  *M* = 15.2 years (in-service) | Perceived knowledge of ADHD  Knowledge of ADHD | None | In-service or pre-service  Age  Gender  Training on ADHD  Teaching experience  Experience of teaching ADHD student  Number of students with ADHD taught | - Teachers completed a single item visual analogue scale asking them to rate their level of knowledge about ADHD, and then a 27-item knowledge test. - Perceived and actual knowledge were moderately correlated (.46), whilst additional training and having taught a student with ADHD were also low/moderately correlated with actual knowledge scores (.24 and.42) - In-service teachers correctly answered 16.4 (SD=4.0) of the 27 actual knowledge items, an average actual knowledge score of 60.7%. Preservice teachers, average number of correctly answered statements was 14.2 (SD = 4.6) of the 27 items, an average actual knowledge score of 52.6%. - In-service teachers rated themselves significantly higher on perceived knowledge about ADHD than did preservice teachers, and in-service teachers scored significantly higher on the actual knowledge questionnaire than did preservice teachers |
| Kypriotaki and Manolitsis (2010)  Greece  *In-service* | ADHD  365 teachers, 420 students  NR  NR  NR  NR | Recognition of ADHD in classroom | None | Student factors:  Grade level (1^st^-3d vs. 4^th^-6^th^)  Gender  Parental education Students’ school achievement  Peer relations  Students’ frequency of participation in group activities  Quality of students’ involvement behaviour in group activities  Peer acceptance  Teacher–child relationship  Teacher–parent cooperation | - Teachers were asked to nominate which students in their classroom they suspected had ADHD, and then completed the ADHD rating scale for each student - 9.1% of the boys and 74.3% of the girls attending Grades 1–3 who were initially identified by their teachers as children with ADHD received scores in the scale above the 85th percentile. In Grade 4-6, 88.4% of girls initially identified by their teachers as having ADHD received scores above the 85th percentile, a significantly smaller proportion of boys (62.5%) in this age group were identified. - Teachers initially identified significantly more boys than girls (χ2(1) = 167.6 p < 0.001), but were more accurate at identifying girls than boys (false alarms more frequent with boys) - Teachers’ judgments about students’ peer relations and teacher–student relationship were the best student-related predictors of children’s scores on the ADHD rating scale-IV |
| Layne et al. (2006)  USA  *In-service* | Internalizing (Anxiety)  453 students  NR  NR  NR  NR | Recognition of Anxiety in the classroom | None | Student factors:  Gender  Grade level  Age  Sub-scale of MASC | - Students completed the MASC and teachers were asked to nominate the three most anxious students in their classroom - Significant differences were found by nomination status, children who were nominated by their teacher were found to have higher scores on all the MASC sub-scales than non-nominated children, except for the harm avoidance subscale. - No significant interactions were found between nomination status and student gender or grade level, indicating that teachers were equally good at identifying anxiety in males/females and older/younger students. |
| Lee et al. (2015)  Germany  *Pre-service* | ADHD  234  NR  87.18% female  NR  NR | Knowledge of ADHD | None | Type of teaching role (general vs. special)  Study progress in university course  Personal experience with ADHD  Training in ADHD | - Teachers completed a survey with 23 knowledge items - Mean knowledge score was 34.87/43 (SD = 3.29), 51.6% correct - Special education pre-service teachers’ knowledge of ADHD (M = 35.81, SD = 3.12) was significantly higher than that of primary education pre-service teachers (M = 34.07, SD = 3.22; t(232) = 4.18, p < .001). The effect size was medium (d = .55). - Pre-service teachers who have learned during their study skills to manage children with ADHD (M = 35.91, SD = 3.18) showed significantly higher knowledge compared to those without such education (M = 34.07, SD = 3.18; t(224) = 4.30, p < .001), medium effect size (d = .56) - No significant difference was found between preservice teachers with personal experience of ADHD (M = 35.33, SD = 3.39), and pre-service teachers without personal experience (M = 34.58, SD = 3.20; t(232) = 1.72, p = .086) regarding their knowledge. |
| Loades and Mastroyannopoulou (2010)  United Kingdom  *In-service* | Externalizing (ODD), Internalizing (SAD)  113  *M* = 38.3  72.57% female  White British (85%) White European (3.5%), Asian (1.8%), Other (2.7%). Not reported (7.1%)  24.8% of sample < 5 years  32.7% of sample > 20 years | Recognition of MHP | Perception of severity of problem  Intention to provide help  Perceived level of concern | Severity of disorder (clinical vs. sub-clinical)  Type of disorder (“behavioural” vs “emotional”)  Teaching experience  Self-rated experience of working with children with mental health problems  Gender (of student) | - Teachers responded to two vignettes (ODD and SAD), one male and one female, at three levels of severity (clinical, sub-clinical and no symptoms), with the gender assigned to the vignettes randomized. - Teachers successfully rated the severity of the vignettes in order: Clinical ODD (M= 2.79, SD = 0.53) vignette rated higher in severity (z=7.22, p <.01) than sub-clinical ODD vignette (M= 2.04, SD = 0.74). Sub-clinical ODD vignette (M= 2.04, SD = 0.74) rated as significantly more severe than problem-free child vignette (M= 0.66, SD = 0.87). - Clinical SAD (M= 2.13, SD = 0.82) rated more severe than sub-clinical SAD (M= 1.28, SD = 0.80). Sub-clinical SAD ((z = )7.84, p < .01) (M= 1.28, SD = 0.80) rated as more severe than problem-free child vignette (M= 0.16, SD = 0.51). - Teachers reported more concern (z= -6.78, p < .01) for clinical ODD (M= 3.63, SD = 0.74) than clinical SAD (M= 2.86, SD = 1.06). - Teachers who completed questionnaire where ODD vignettes presented as boys and SAD vignettes presented as girls were significantly more accurate in terms of problem recognition than teachers who completed ODD vignettes presented as girls and SAD vignettes presented as boys. |
| Miranda Padilla et al. (2018)  Colombia | ADHD  62  *M* = 45.97  98.39% female  NR  80.65% of sample > 10 years | Knowledge of ADHD | Perceived self-efficacy in teaching children with ADHD | None | - Teachers completed the 36 item KADDS, which has three subscales: symptoms/diagnosis of ADHD, general information about the nature, causes and repercussions of ADHD and treatment of ADHD - Teachers correctly answered slightly fewer than half the items in the questionnaire (48.52%). The most correct answers were on the symptoms/diagnosis subscale (69.35%), followed by the treatment subscale (45.30%) and the general information subscale (38.60%) - On a 7-point scale of self-efficacy, a score of 4 was the most common (27.42%), followed by 3 (25.81%). Only a small percentage of teachers (6.45%) felt fully prepared to teach children with ADHD. |
| Moldavsky et al. (2013)  United Kingdom | ADHD  496  *M* = 40.5  85% female  NR  58% of sample > 10 years | Recognition of ADHD | Need for referral  Rating of preference of intervention  Factors affecting decision to refer | Sub-type of ADHD (inattentive vs. combined)  Gender (of student)  Teaching experience | - Teachers responded to one of four vignettes: 2 describing the behaviour of a 9-year-old girl or boy with inattentive subtype of ADHD, and two related to a 9-year-old girl or boy with combined subtype, with a 6-question survey - 33% of teachers identified ADHD in the inattentive boy vignette, 59% in the combined, 33% in the inattentive girl and 54% in the combined. - The vignette of the combined subtype was associated with greater recognition of ADHD (OR = 2.81; 95% CI 1.93–4.08; p < .001). - The likelihood of teachers considering a need for referral to specialist services, was 5.9/10 for a boy with combined sub-type and 5.4/10 for the other three conditions. - Intervention approaches strongly endorsed by teachers were (learning support: 100%; work with parents: 97%; behavioural interventions: 95%). Only 13% of teachers thought that medication might be helpful, particularly for the combined subtype of ADHD for both genders |
| Muanprasart et al. (2014)  Thailand | ADHD  201  *M* = 45.01  85.6% female  NR  *Mdn* = 25 years | Knowledge of ADHD | None | Age  Personal experience with ADHD  Source of knowledge on ADHD | - Teachers completed the 36 item KADDS, which has three subscales: symptoms/diagnosis of ADHD, general information about the nature, causes and repercussions of ADHD and treatment of ADHD - Highest knowledge scores were on the signs/symptoms and diagnosis subscale (62.7% getting more than 50% correct), general information and treatment subscales were lower (12.9% and 12.4% getting more than 50% correct). - Younger teachers were more likely to get a perfect score on the KADDS (adjusted OR = 3.717, p = 0.041) - Familiarity with those with ADHD was associated with higher knowledge scores (adjusted OR = 3.218, p = 0.003) |
| Neil and Smith (2017)  United Kingdom  *In-service* | Internalizing (Anxiety)  51 teachers, 1346 students  NR  NR  NR  NR | Recognition of anxiety  Recognition of somatic symptoms | None | Anxiety symptoms of students (child and parent reported) | - Students and their parents completed the SCAS and CDI. Teachers completed a rating form, asking them to rate each child in their class on a scale of 1 to 5 as to how anxious they had been in the past two weeks - Small but significant positive association between children’s self-reported SCAS scores and teachers’ anxiety ratings (rs (1,333) = .14, p < .001). Children assigned a teacher anxiety rating of “1,” and those assigned a “2” rating both independently had significantly lower SCAS scores than children assigned a rating of “3,” “4,” or “5.” - Children nominated by their teacher as having debilitating levels of anxiety did not have significantly higher scores on the self-reported SCAS (M = 31.76, SD = 19.43) than those not selected (M = 32.25, SD = 17.81; t(1,333) =−.226, p = .82). However, children identified as having high levels of somatic symptoms by their teacher did have significantly higher self-reported somatic scores (M= 24.68, SD = 16.49) than those who were not identified as such, although the effect size was modest (M= 20.02, SD= 14.51; t(1,325)=−2.45, p = .014, d = .30). |
| Ní Chorcora and Swords (2021)  Ireland  *In-service* | Internalizing (Anxiety, Depression)  356  NR  83.1% female  NR  *M* = 14 years | Recognition of internalizing disorder | Concern expressed for internalizing disorder  Confidence in helping  Intention to help | Gender  Teaching experience  Personal experience with ADHD | - Teachers responded to three vignettes: one describing GAD, one describing depression, and a control, non-clinical vignette. The vignettes were counter-balanced for gender. Questions following the vignettes were around teachers recognition of MHPs, a measure of their concern, and their likelihood of helping them, and what help-giving actions they would take. - **Recognition of GAD in vignette:** 3.9% of teachers reported that the child in the vignette had an ‘anxiety disorder’. A further 84.3% mentioned ‘anxiety’ in some form in their response. The remaining 11.8% did not identify the child with GAD as experiencing anxiety. - **Recognition of depression in vignette:** 71.3% correctly identified the description of clinical depression by responding with the words ‘depression’/’depressed’/’depressive’ in their answer. Two participants (0.6%) specified the child as having a ‘depressive/mood disorder’ or ‘clinical depression’. The remaining 28.7% did not perceive the child as experiencing depression. - Teachers were significantly less concerned for the ‘non-clinical’ vignette character (M= 2.05, SD=.036) compared with both the child in the GAD vignette (M= 3.75, SD= 0.24) and the child in the depression vignette (M = 3.78, SD= 0.25). There was no significant difference in the amount of concern reported for across the two clinical vignettes (GAD, depression). - Gender (β = .137) and exposure to mental illness (β = .113) were significant in that women and teachers with greater exposure to MHPs expressed greater concern for students with internalizing disorders (R2 =.032, F(3, 350) = 3.861, p=.01.) - While concern and confidence were associated with greater willingness to help, more years of teaching experience were associated with less willingness to help. Teachers’ confidence in their ability to help was the strongest predictor of their likelihood to help (R2 = .170, F(5, 348) = 14.231, p < .001) |
| Ohan et al. (2008)  Australia  *In-service* | ADHD  140  *M* = 42.33  85% female  NR  *M* = 19.76 years | Knowledge of ADHD | Need for referral  Benefit of intervention for student  Prediction of future behaviour | Gender  Teaching experience  Number of students with ADHD taught  Number of students who teacher had sought support for | - Teachers responded to a 20-item self-report survey and 10 vignettes describing either boys or girls with inattentive and hyperactive-impulsive behaviours, with questions - Knowledge: Mean correct score was 76.34% (median = 14.00 (73.68%)) - No significant impact of gender, teaching experience or number of children taught with ADHD on knowledge - Teachers with high knowledge were significantly more likely than those with low knowledge to endorse the need for (Cohen’s d = .67) and seek professional assessment services (Cohen’s d = .64) - Teachers with high and average ADHD knowledge were more likely than those with low knowledge to acknowledge the likelihood of ADHD impact on the child’s classroom (high knowledge, Cohen’s d= .75; average knowledge, Cohen’s d= .47) and peer relationships (high knowledge, Cohen’s d = 1.03; average knowledge, Cohen’s d = .66). In addition, teachers with low ADHD knowledge were significantly more likely to report that they would be able to handle the child’s problems without assistance than were teachers with high (Cohen’s d = .63) and average (Cohen’s d = .54) knowledge. |
| Sciutto et al. (2000)  USA  *In-service* | ADHD  149  *M* = 40.8  89.93% female  NR  *M* = 12.57 years | Knowledge of ADHD | Confidence in ability to teach child with ADHD | Age  Education level  Teaching experience  Number of special education classes taken Number of students with ADHD taught | - Teachers completed the 36 item KADDS, which has three subscales: symptoms/diagnosis of ADHD, general information about the nature, causes and repercussions of ADHD and treatment of ADHD - Mean total knowledge score was 17.21/36 (SD=6.70) - Scores on the symptoms/diagnosis subscale of the KADDS were significantly greater than scores on both the treatment, (F(1,148) = 158.61, p < .001, d = 2.07) and general information subscales, (F(1,148) = 194.73, p < .001, d = 2.29) - Endorsed significantly fewer misperceptions on the symptoms subscale of the KADDS than on both the general, (F(1,148) = 59.18, p < .001, d = 1.27), and treatment subscales, (F(1,148) = 27.19, p < .001, d= 0.86.) - No association found between overall knowledge of ADHD and various teacher characteristics, including age, education level, and number of special education classes taken. An association was found between knowledge and teachers’ confidence in their ability to effectively teach an ADHD child, (r(145) = .29, p <.001.) and smaller correlations with the number of ADHD children taught, (r(128) = .22, p = .011) and years of teaching experience, (r(142) = .18, p = .029.) |
| Splett et al. (2019)  USA  *In-service* | Internalizing, Externalizing  153  NR  90.2% female  75% European American  10.46% African American  2.61% Hispanic  0.65% Asian  40.52% of sample ≥ 10 years | Recognition of MHP | Rating of severity of MHP  Rating of concern  Referral for help | Severity of MHP (moderate vs. severe) | - Teachers responded to one of two vignettes (ODD and SAD), one male and one female, at three levels of severity (clinical, sub-clinical and no symptoms), with the gender assigned to the vignettes counter balanced. - **Accuracy of problem identification:** No significant difference in accuracy for the severe versions of the internalizing or externalizing, and teachers rated the severe versions as more severe than the moderate versions (externalizing behaviour problems Z = − 7.94, p < .001, r = 0.65; internalizing behaviour problems Z = − 7.94, p < .001, r = 0.65 ). For the moderate symptom vignette, a significant difference on teacher accuracy was found between problem types : teachers’ correct identification was higher for the externalizing vignette (91%, n = 139) than for the internalizing (73%, n = 111) vignette. - **Rating of severity:** Regardless of problem severity described in the vignettes, teachers rated externalizing behaviour as being more serious and indicated a higher level of concern than for internalizing behaviour - Teacher level of concern were associated with greater odds of referring children on to school based mental health services in both the severe externalizing and internalizing conditions (Externalizing OR = 4.561, internalizing OR = 3.973). For the moderate conditions, teacher concern was only associated with greater referral to school based mental health service for the internalizing scenario (OR=3.463), in the externalizing scenario greater concern was associated with greater referral to community mental health practitioners (OR = 3.874). |
| Topkin and Roman (2015)  South Africa  *In-service* | ADHD  200  *M* = 43  89% female  33.8% Black  40.9% Coloured  1.5% Indian  29.5% sample = 0-12 years  28.9% sample = 13-20 years  41% of sample > 21 years | Knowledge of ADHD | Perception of classroom management of ADHD  Training received  Access to resources on ADHD | None | - Teachers completed the 36 item KADDS, which has three subscales: symptoms/diagnosis of ADHD, general information about the nature, causes and repercussions of ADHD and treatment of ADHD, and 13 items on management - Knowledge: Total mean correct 45%, 31% “don’t know, 22% incorrect - Highest mean correct answers on the General Associated Features subscale (65%), lowest on the symptoms and diagnosis subscale (36%) - Teachers were mainly supportive of using educational interventions (97%) and classroom rules (91%) for classroom management - The least supported of the classroom management interventions were: time given for tests (58.4%); and ignoring the disruptive behaviour (66.7%) |
| Vereb and DiPerna (2004)  USA  *In-service* | ADHD  47  NR  94% female  NR  *M* = 13 years | Knowledge of ADHD Knowledge of treatments for ADHD | Attitude towards treatments for ADHD (behaviour management/medication) | Teaching experience  Training in ADHD | - Teachers completed the KARE, which included questions on knowledge of ADHD (31), knowledge of treatments commonly used for ADHD (12), medication acceptability (5), and Behavior Management Acceptability (5) - Knowledge scores ranged from 14 to 27 items correct out of a possible 31 items (M = 21.57, SD = 3.68). - Years of teaching experience with students with ADHD was significantly related with ratings of medication acceptability. Teachers’ participation in training regarding ADHD was positively correlated with their knowledge of ADHD, acceptability ratings of medication, and acceptability ratings of treatments |
| Walter et al. (2006)  USA  *In-service* | General  254  *M* = 41  82% female  NR  *M* = 15 years | Knowledge of MHPs | Ranking of most important MHPs  Preferences for training  Attitudes towards provision of MH services in schools  Self-efficacy in managing MHPs | Teaching experience  Teacher level of education | - Teachers responded to a survey asking them about their knowledge of MHPs, their confidence in dealing with MHPs, their preferences for mental health services in schools and ranking of MHPs by importance. - **Most important mental health issue:** 48% of teachers endorsed disruptive classroom behaviour (e.g., getting out of a seat, talking out of turn, arguing, failing to comply with rules and requests) as the biggest problem, followed by students who are not motivated to learn (15%), students who are disrespectful of authority (13%), bullying/cliques (9%), and disruptive playground behaviour (8%). - **Knowledge:** Mean percentage of correct answers on MH knowledge was 65. Most correct answers were: ‘‘most children and adolescents with psychiatric disorders receive no treatment’’ (true); ‘‘both inherited and environmental factors cause major depression in children’’ (true); ‘‘the key symptoms of major depression are sad mood and/or loss of interest or pleasure in most activities’’ (true); the least correctly answered questions were “The key symptoms of ADHD are inattention, hyperactivity, and irritability. (False), “the most common psychiatric disorder in children and adolescents is depression.” (False), “Most children with untreated oppositional defiant disorder become juvenile delinquents. (False)” - **Confidence in dealing with MHPs:** The overall mean score on the self-efficacy scale was 1.82 on a 3-point scale (between ‘‘not at all confident’’ and ‘‘somewhat confident’’) |
| Whitley and Gooderham (2016)  Canada  *Pre-service* | Internalizing (Depression, Anxiety), Externalizing (ODD, ADHD)  186 (55% completed child version of vignettes)  *M* = 26.5  73% female  NR  NR | Recognition of MHPs | Self-efficacy managing MHPs  Expectations of student progress  Rating of problem severity  Rating of intervention priority  Teaching strategies used  Attributes for behaviour | Personal experience with MHPs  Experience working with students with MHPs | - Teachers responded to four vignettes, depicting children with academic difficulties plus depression or ADHD, and no academic difficulties plus ODD or anxiety. A survey followed with questions around recognition of MHP, perceived severity, perceived self-efficacy in dealing with MHPs, student priority for intervention, and what strategies would work with the student - **Self-efficacy:** Teacher efficacy was lowest with respect to working with ODD vignette and highest when working with anxiety vignette. - **Expectations**: Expectations were lowest for ODD child and highest for anxious child - **Problem severity:** Most severe was rated as the depression plus academic concerns vignette and lowest for the vignette depicting ADHD plus academic concerns. - **Intervention priority**: The vignette depicting depression plus academic concerns was seen as a top priority and the vignette depicting ADHD plus academic concerns as the lowest. - **Problem recognition:** 38.50% of sample identified the depression vignette as being caused by depression, 89.6% recognized anxiety, 84.40% recognized ADHD and 47.90% recognized ODD. |
| Whitlock et al. (2020)  United Kingdom  *In- service and pre-service* | ASD  289  *Mdn* = 31  94.1% female  NR  *Mdn* = 6 years | Recognition of ASD in vignette | Likelihood of seeking support for student | Phenotype of disorder (“female”, “male”, separation anxiety, ADHD)  Gender (of student)  Personal experience with ASD  Training in ASD  Number of students with ASD taught | - Teachers responded to four vignettes, one depicting the male-ASD phenotype, one depicting the female-ASD phenotype, one of SAD and one of ADHD. Gender was counter-balanced amongst the vignettes. A survey followed with questions around recognition - Teachers were more likely to identify ASD in males than females and were more sensitive to the male phenotype in comparison to the female phenotype of ASD. For the vignette phenotype, being presented with a male phenotype resulted in an average 14.53 increase (on a scale ranging from 0 to 100) in likelihood rating in comparison to the female phenotype. - For the male ASD phenotype vignettes, having worked with four or more children with ASD was predictive of sensitivity to recognizing ASD. |
| Woyessa et al. (2019)  Ethiopia  *In-service* | ADHD  206  62.6% of sample > 40  50.5% female  NR  42% sample > 16 years | Knowledge of ADHD | None | None | - Teachers completed the 36 item KADDS, which has three subscales: symptoms/diagnosis of ADHD, general information about the nature, causes and repercussions of ADHD and treatment of ADHD - ”Misconceptions” in knowledge of ADHD: Mean percentage of teachers with at least one misconception (incorrect answer): general subscale: 76.2%, symptoms/diagnosis subscale: 62.7%, treatment subscale: 81% |
| Parent Samples | | | | | |
| Dang et al. (2021)  Cambodia, Vietnam | General (SAD, ODD, SSD, Depression, Trauma)  357  *M* = 35.44  100% female  NR | Recognition of MHPs | Attributes for causes Attitudes towards treatments | Country (Cambodia vs. Vietnam) | - Mothers completed the CMHLQ - Recognition was low, correct identification ranged from 17% (for Oppositional Defiant Disorder) to 35% (for Trauma-Related Mental Health Problems) - Cambodian mothers recognizing trauma-related problems as ‘‘mental health’’ more frequently than Vietnamese mothers - Cambodian mothers had lower levels of endorsement of Skills Development as effective interventions for the vignette mental health problems but higher levels of endorsement of Punishment and Reward than the Vietnamese mothers - Vietnamese mothers having higher levels of endorsement of biological factors and lower levels for ‘‘bad character’’ than the Cambodian mothers |
| Huang et al. (2019)  Australia | General  4983 children  and their parents  NR  NR  NR | Recognition of MHPs | None | Income  Age (of child) | - Parents completed SDQ measure for their children and also answered a question that asked them to report any ongoing emotional (anxiety or depression), behavioural (attention-deficit hyperactivity disorder) or developmental (autism, Asperger’s, or other autism spectrum problems) problem. - Parent recognition was lowest amongst the lowest income quartile (11.5% of children who scored in clinical range of SDQ not perceived to have a mental health problem) - In 4-7 age range, 1.4% of parents perceived a MHP in their child, yet 9.3% scored in the clinical range of the SDQ - In the 8-11 age range, 4.9% of parents perceived a MHP in their child, and 9.0% scored in the clinical range of the SDQ |
| Lowinger (2009)  USA | General  157  29.3% sample > 45  71.3% female  NR | Recognition of MHPs | Perception of severity of problem  Perception of commonality of problem  Preference for treatment (source, type and provider)) | Type of MHP internalizing vs. externalizing | - Parents responded to one of two vignettes, one describing a male with externalizing symptoms, and one describing a male with internalizing symptoms. A survey followed with questions around whether they viewed the symptoms as a problem, attitudes about severity and willingness to have the child referred to professional help. - **Severity of problem:** *Externalizing vignette:* 91.8% of the parents strongly agreed or agreed that it was a problem. *Internalizing vignette:* 88.0% of parents strongly agreed or agreed that it was a problem. - **Seriousness of problem:** Externalizing behaviour (M=1.54, SD=.74) was rated as significantly more serious than internalizing behaviour (M=1.79, SD=.80) (F (1,154) = 3.98, p<0.05) - **Willingness to seek treatment:** For the externalizing problem, 87.6% either strongly agreed or agreed while for the internalizing problem, 75.9% either strongly agreed or agreed that help should be sought - Parents were significantly more likely to refer a child with externalizing behaviour to school psychologist, hospital, community organization or youth program than a child with internalizing behaviour. School psychologist and counselling was the top preference of parents (F (4, 151) = 32.72, p<.00). |
| Villatoro et al. (2018)  USA | General  432  *M* = 38.39  78.94% female  72.4% Ethnic minority | Recognition of MHPs (in own child and hypothetical child) | Parent stigmatising attitudes  Perception of seriousness | Gender  Income  Ethnicity  Familiarity with MHPs  Personal experience with MHP’MHPs  Gender (of child)  Family cohesion  Type of MHP (social anxiety vs bipolar) | - Parents responded to both a vignette scenario (one depicting a male with SoA, and one depicting a female with BD) and a survey with questions that asked them to identify if their own child had a MHP, stigma and a variety of socio-demographic factors. Children completed a survey which asked them if they thought they had a MHP. - Fewer than one-third of parents (29.1%) in the total sample recognized a mental health problem in their preadolescent child, this jumped to 58% in subsample of parents of children with greater number of MH symptoms ( >9), although the concordance with child reported MHP was lower - The more social distance parents desired for their child, the less likely they were to recognize a problem (OR: 0.48, p<0.05). This was particularly true in the high-symptom sub-sample. - Family history, familiarity, parent-reported symptoms, and family income greater than 75K were significantly associated with high odds of problem recognition (OR: 2.23, 1.39, 1.36, 2.91, p<0.05) - Significantly fewer parents rated a social anxiety vignette (David) as a concern compared to a bipolar vignette (Julia) and reported lower social distance desire for the same. The more serious they rated a vignette characters problem, the more likely they were to recognise it (Julia OR: 1.91, p<0.01; David OR: 3.76, p <0.001). - Parental Stigma (desire for social distance) was associated with reduced problem recognition in their own children but not in unrelated children described in vignette |
| Mixed Parent/Teacher samples | | | | | |
| Bevaart et al. (2012)  Netherlands | General  17,511  *n(parents)* = *8,114*, *n(teachers) = 9,397*, *n(children) =10,951*  NR  NR | Recognition of MHPs | Perceived need for help | Ethnicity | - Teachers and parents completed the SDQ for their own child or children they taught. They then responded to a survey with questions that asked them to identify if the child had a problem with emotions, concentration, behaviour or the ability to get along with other people and their perceived need for care (“Do you think the child needs professional help?”) - 63.1% of parents of screen positive children (above 90^th^ percentile, not currently in treatment) on the SDQ perceived their child to have emotional or behavioural problems. - 87.2% of teachers perceived a screen positive child to have emotional or behavioural problems. |

*ASD = Autism Spectrum disorder, ODD = Oppositional Defiance Disorder, SAD = Separation Anxiety Disorder, GAD = Generalized Anxiety Disorder, ADHD = Attention Deficit Hyperactivity Disorder, BD = Bipolar Disorder, SoA = Social Anxiety Disorder, SSD = Somatic Symptom Disorder, MHP = Mental health problem, MH = Mental health, OR = Odds Ratio, SDQ = Strengths and Difficulties Questionnaire, NR. = Not reported, M = mean, Mdn = median, SD = standard deviation, CI = confidence interval, in-service= teacher in employment in a school setting, pre-service=teacher still undergoing their teacher training, CMHLQ = Child Mental Health Literacy Questionnaire, KADDS = Knowledge of Attention Deficit Disorder Scale, CDI = Child Depression Inventory, MASC = Multidimensional Anxiety*Scale*for Children, SCAS = The Spence Children's Anxiety Scale, KARE = Knowledge of ADHD Rating Evaluation*
